# Measuring trust with the Wayfinding Task: Implementing a novel task in immersive virtual reality and desktop setups across remote and in-person test environments

**DOI:** 10.1371/journal.pone.0294420

**Published:** 2023-11-28

**Authors:** Michael F. Clements, Larissa Brübach, Jessica Glazov, Stephanie Gu, Rahila Kashif, Caroline Catmur, Alexandra L. Georgescu

**Affiliations:** 1 Department of Psychology, Institute of Psychiatry, Psychology and Neuroscience, King’s College London, London, United Kingdom; 2 Human-Computer Interaction (HCI) Group, Julius-Maximilians University of Würzburg, Würzburg, Germany; Politecnico di Torino, ITALY

## Abstract

Trust is a key feature of social relationships. Common measures of trust, questionnaires and economic games, lack ecological validity. Hence, we sought to introduce an immersive, virtual reality (VR) measure for the behavioral assessment of trust across remote and in-person settings, building on the maze task of Hale et al. (2018). Our ‘Wayfinding Task’ consists of an interconnected urban environment for participants to navigate on the advice of two characters of differing trustworthiness. We present four studies implementing the Wayfinding Task in remote and in-person testing environments and comparing performance across head-mounted display (HMD)-based VR and desktop setups. In each study, the trustworthiness of two virtual characters was manipulated, through either a fact sheet providing trustworthiness information, or a behavior-based trustworthiness manipulation task termed the Door Game, based on Van der Biest et al., 2020. Participants then completed the Wayfinding Task. Overall, we found that participant behavior in the Wayfinding Task reflected the relative trustworthiness of the two characters; in particular, the trustworthy character was approached more often for advice, reflecting data from our Door Game. We found mostly null results for our novel outcome measure, interpersonal distance. Remote testing successfully achieved these effects. While HMD-based VR and desktop setups both showed these effects, there was a stronger effect of trustworthiness in the HMD VR version of the task. These results have implications for the measurement of trust in behavioral settings and the use of remote and VR-based testing in social experiments.

## Introduction

Trust is a facet of interpersonal communication which affects many aspects of our lives as social beings. From when it was first conceived experimentally, trust research has been considered of relevance to such topics as mental illness and wider societal problems [1, 2]. Trust affects social norms and preferences [3] and plays a key role in the intersection of fields, such as the integration of power dynamics in systems of mental health [4] and metascience [5]. On a personal level, trust relates to developing relationships [6], from strangers [7], to professionally, and with partners and family [3, 8]. Thus, trust is of importance to researchers involved in studying human dynamics.

Where trust may be most salient experimentally is its role in interpersonal communication between pairs, or groups, of individuals. From the perspective of interpersonal communication, trust is a key feature of social relationships and shapes our behavior towards others [1, 9]. Definitions of trust vary in the literature [10, 11] but classically involve certain core components, commonly predictability of the trustee’s behavior across repeat performance and motivational relevance alongside some form of vulnerability on behalf of the trusting individual [1]. Given the relevance of trust to interpersonal interactions; its reflection in behavior in naturalistic settings; and the increasing body of research integrating studies of interpersonal trust with emergent technology, specifically virtual reality (VR) [12–14] there is a need for studies and designs which can experimentally replicate and measure interpersonal trust and trustworthiness in a reliable and valid manner.

To design this type of study, researchers must consider which factors can influence one’s trust in others. In a naturalistic environment, trust can be developed in the process of learning about and testing your relationship with an individual [15, 16]. We can also infer trustworthiness from others based on everyday behaviors [17]. However, this is not always ideal for establishing the basis of an experiment. Indeed, stable perceptions of trust can form immediately on first impression, such as by judging the trustee’s facial cues [18–21]. Trust priming has been shown to lead to different outcomes in trustworthiness from manipulations as simple as using the word ‘partner’ or ‘opponent’ during the introduction of an exercise [22]. In short-term relationships, one of the strongest predictors of trustworthiness is access to social information, such as knowledge about another’s character [21]. This access to information also shapes choice behaviors such as preferences in individuals [23]. Thus, there is a basis for framing and presenting trust as social information in an experimental setting. However, as trust is pervasive in interpersonal relationships, it can be hard to measure trustworthiness appropriately.

The simplest method for measuring trustworthiness levels is via questionnaire; for example, asking participants explicitly how much they trust a given individual. Hale et al. [24] highlight that such responses are sensitive to demand characteristics [25] and may reflect participants being trusting in general, rather than the trustworthiness of a specific other [26]. Given the relevance of trust research to economic outcomes, behavioral alternatives for trustworthiness measurement are commonly found in the form of economic games. Economic games, like the investment game [27], are frameworks sensitive to differing levels of trustworthiness between characters. They evaluate trust relationships through the amount of money, or points, that one is willing to reciprocally invest in another interaction partner. Participants may pledge a specific amount to one character, which is then increased when it is sent to the character. This character may then send back a portion of the increased investment, or even nothing at all. The participants’ trust in each character is then indexed by the amounts which they continue to send to each character, while expecting a return.

While an improvement over questionnaires in terms of ecological validity, these types of judgements suffer difficulties in experimental settings. Investment games suffer from a similar shortcoming to trust questionnaires, where they were originally designed to reflect generalized trust; one’s propensity to trust any given person, rather than the levels of trust one may have in different individuals [26, 28, 29]. As these games also reflect generalized trust in settings where characters have different levels of trustworthiness [24] this makes these games inappropriate tools for only looking at the comparisons between characters. Additionally, these manipulations may not be generalizable to common social, non-economic settings. A value statement, such as investment amount, does not appropriately gauge the predictability aspect of trust [1, 30] which influences human-agent interactions [31]. The need for an investment strategy to produce greater returns can also interfere with the measure of trust provided by the initial investment [32]. Therefore, trust researchers may wish to design measures of trust which avoid financial value judgements altogether.

The design used in the present work is therefore based on the ask-endorse paradigm [33, 34]. Two characters are introduced to the participant via a manipulation which should be expected to induce differential levels of trust. As an example, in previous versions, one character lied while one told the truth [33]. The participant is then placed into a scenario where they can question each character about a novel situation, and then ultimately make a decision on how to act based on their advice. Hence there are two measures of trustworthiness founded in behavior; both who is asked, and whose advice is endorsed through the participants’ final decision. Importantly, the character’s actual trustworthiness is not fed back to the participant in the same way as a financial return in an economic game; instead, these measures provide a behavioral proxy for the researcher to quantify the participant’s trust in the characters. While the original research was focused on children, the ask-endorse paradigm has been successfully replicated in adults, in particular the maze task developed by Hale et al. [24]. Importantly, this task was constructed in VR, which offers high ecological validity, as in confederate studies, without suffering the same shortcomings of variability and lack of control that can lead to confounds from facial cues or other features; thus making it an ideal environment for the modelling of social interaction, which requires both tight control and high ecological validity to maintain face validity. This synthesis with a behavioral measure of trust thus allows, in theory, for a measure of trust with high face validity.

Hale’s maze task consisted of a series of rooms with ‘holograms’ of characters in each, where participants could approach either character and ask for advice before deciding on which way to proceed. Overall, it was found that participants not only asked the trustworthy characters more frequently for advice, but followed the advice of the trustworthy character more often (showing that they endorsed them more frequently).

However, Hale and colleagues’ maze task demonstrated variable sensitivity to their manipulations of trustworthiness. In their first two studies, they included non-verbal cues linked to trustworthiness, such as eye contact, which may have contributed to confounding experiences like rapport instead of trust [24]. In their third study outside of immersive (Head Mounted Display, or HMD-based) VR, they controlled for these factors, but observed much lower effects, potentially due to the less immersive setting. Despite having improved ecological validity compared to other studies, the setting and cover story for these studies were rather minimalistic. In all settings, also, the characters were not present in the environment. They appeared as holograms in the first two studies, which may be less ecologically valid a scenario, and were only contactable via phone call in the third. Hence there is a need to validate a more ecologically valid version of the maze task as a measure of trustworthiness, and to examine the role of VR in its implementation.

One key argument that Hale et al. [24] put forward was that the ask-endorse approach can represent an ecologically valid scenario of trust measurement; giving the example of asking a passer-by for directions, and trusting whether to follow their advice based on limited experience. We build on their scenario, framing our characters as part of an open-plan environment made to look like a city instead of identical rooms, which participants were tasked to navigate. Our Wayfinding Task comprises a series of decision points within this city environment (functioning as crossroads). At each decision point participants encounter two characters and can consult one or both regarding which direction to travel. Additionally, alongside the behavioral parameters examined by Hale (which character’s advice was followed, who was asked for advice more frequently, and who was asked for advice first)prior research has shown conflicting evidence that trust, as manipulated by trust games, is associated with closer [35] or further interpersonal distance [36]. We hence propose a two-tailed hypothesis regarding interpersonal distance between the participant and character(s) as an additional measure of trust, and predict a one-tailed hypothesis showing our other aforementioned behaviors to more frequently occur for the trustworthy character. Ultimately, while incorporating the above methodological considerations, we present our implementation of this new Wayfinding Task as a measure of our characters’ trustworthiness.

To establish different levels of trustworthiness in our characters, it is important to include some form of manipulation. In the present work, we use two manipulations which are intended to induce different levels of trustworthiness while requiring no monetary valuations to be assigned by the participants. In Study 1, we used a minimal design, presenting trust-associated social information using fact sheets regarding our characters. Our aim by presenting socially salient information was to inform participants of one of the core aspects of trust, suggesting how likely our characters would be to prevent negative outcomes for the participant during their experience [2] in line with how access to social information has previously indicated trust preference in adolescents [23]. The fact sheets were presented in the style of the interviews used in Hale et al. [24]’s Study 1 and 2, but transcribed so as not to introduce any possible confounds from vocal cues. In Studies 2 onwards, we implemented an adaptation of a task called the Door Game which has been validated as a task for trust manipulation [37]. In this task, participants are presented with the advice of each character in turn, and then must select which door to enter, receiving points-based feedback. One character, designed to be trustworthy, presents advice which would always grant the participant points, and the other gives advice seemingly at random. Thereby our participants may deduce which character’s advice is ‘accurate’, and therefore who is more trustworthy, before being placed into the VR Wayfinding Task where they can consult the characters for advice freely and choose whether to endorse these responses.

One potential issue with not collecting quantitative measures during our trustworthiness manipulations is that if no effect of the manipulation is found on the dependent variables measured during the Wayfinding Task, we cannot be sure whether this is because the Wayfinding Task is insensitive to our manipulation, or alternatively whether the manipulation itself is ineffective. To verify whether the manipulation was effective we included a trust-related version of the Implicit Association Test (IAT) [38]. This version of the IAT measures trustworthiness more implicitly than questionnaires, and has been used for virtual characters in assessment of the Door Game [37]. While it continues to lack ecological validity as compared to the Wayfinding Task and does not allow the measurement of specific trust behaviors, this makes it a useful tool for confirming whether our trustworthiness manipulations may have been successful. From Study 2 onwards, our Door Game also provides measures from which we can observe whether it is likely to have manipulated trust. This includes the number of times the participants have followed either characters’ advice, and participants’ reaction times in selecting a door following the advice of either character.

In addition, we manipulated numerous methodological factors across our design, both to address concerns of experimental design raised by Hale and colleagues; and to further expand our work towards an ecologically valid measurement of trust, by changing the design of our trust manipulations, controls for our measurements and comparisons across groups. Another of Hale et al. [24]’s aims, relating to their third study, was to demonstrate the suitability of their maze task for traditional laboratories without VR equipment. However, their desktop adaptation came with difficulties. Their trustworthiness manipulation used an investment game, and the maze task proper was carried out without the characters themselves being present. Instead, they were only present as audio who could be ‘called’ when needed. Although the trustworthy character’s advice was followed more often, 42% of participants stated they relied on audio cues to inform their decision rather than their trust in each character [24]. However, it is important to keep in mind that this behavior is not attributable to voice cues alone, as the voices were counterbalanced for each character. The authors postulate that this audio presence rather than an embodied character may be less socially salient, and hence claim that this simplified task is less suitable than their immersive VR alternative. However, given that Hale et al.’s immersive VR version differed from this simplified audio version in several ways, this still leaves the question of whether traditional computer setups are capable of replicating the behavioral effects found when using immersive VR setups. It is argued that the realistic responses produced by immersive VR setups are the result of feelings of immersion [39] but also that this immersion effect will be stronger in an environment with more perceptual input, for example a head mounted display (HMD) compared to a desktop setup [40]. Hence it remains to be seen whether the behavioral effects observed in the maze task are maintained in the low-fidelity environment of the standard screen and keyboard. To this end, we compare in Study 4 the results found in both HMD and desktop implementations of our maze task. To expand on the aims of making such research accessible, and in light of research challenges posed by COVID-19, we also examine the efficacy of using our Wayfinding Task to measure trustworthiness both remotely and in-person throughout our studies (Table 1).

**Table 1:** Differences in study procedure.

Procedure	Study 1	Study 2	Study 3	Study 4
**Trust Manipulation**	Fact Sheet	Door Game	Door Game	Door Game
**Modality**	VR	VR	Desktop	VR/Desktop
**Location**	Remote	Remote	Remote	In-Person

Overall, we aimed to examine the validity of this Wayfinding Task as a behavioral measure of trustworthiness and its feasibility in remote and in-person environments, using desktop setups and HMD-based VR. We examine behavior in the context of four dependent variables, three of which are facets of the ask-endorse paradigm. These include two ‘asking’ variables (which character was asked first, and who was asked more frequently overall; which represent specific and generalized trust, respectively [24]) and a novel outcome measure, interpersonal distance. We also employ the IAT and data from our Door Game (where applicable) as confirmatory measures regarding our trust manipulation.

## Stimulus selection

When designing stimuli for the characters, there are important considerations to take into account. While there is evidence that vocal cues such as pitch, accent, and hesitations in speech are related to trustworthiness [41, 42], they could also affect perceptions of capability [43]. To avoid these cues, and the effect they may have on results, we used piloting to match potential voices on different qualities. We also did the same for the character models implemented in the maze, as people’s facial appearances can produce stable impressions of trustworthiness [18–21], similarly to social information [22, 44]. As such, we selected characters from the Microsoft Rocketbox virtual avatar library (https://github.com/microsoft/Microsoft-Rocketbox) who had previously been shown to be emotionally neutral in their default expressions [45].

Additionally, as this work was to form the basis for our continued study, our measuring of trust was standardized against previous metrics by use of questionnaires. As this selection process occurred outside of VR space, there was minimal conflict with the desired ecological validity, and with the design of our selection being simple ratings of artificial characters with no predetermined outcome we also avoid potential biases regarding social norms and demand characteristics which may confound questionnaire data [24].

## Methods

### Participants

Our pre-study recruited fifteen participants via word of mouth (13 females, M_*age*_ = 32.40, SD_*age*_ = 7.87) who were offered entry into a prize draw. The study was granted ethical approval by King’s College London’s Research Ethics Committee, registration number MRSU-20/21-21188. Ethical standards herein conform with the declaration of Helsinki, and participants provided informed written consent to take part.

### Procedure

We selected the four characters from the Microsoft Rocketbox virtual avatar library who were of a similar demographic to the characters in Hale’s third study (female, white, and plain-clothed; adult 01, 08, 12, and 17 in the Rocketbox library). By matching our characters on demographics, this helped in controlling for the effect of participant demographic, such as gender or culture, on trust [46, 47]. We similarly recorded six female, Southern English voices reading from a script of directions, from people of the same demographic recruited from peers of the researchers. Rocketbox characters were imported into Unity and had snapshots taken of their in-engine appearance.

Participants gave responses on rating scales for each characters’ friendliness, trustworthiness, intelligence, and confidence. These qualities have been used previously to rate this type of stimuli [48]. Qualities other than trustworthiness were included so participants would not focus solely on trustworthiness and to aid in selection later. Participants rated both the faces and voices on the same characteristics. Ratings were conducted using a 0–100 slider scale ranging from ‘Strongly Disagree’ to ‘Strongly Agree’ on statements adapted for each quality, for example, “This person seems trustworthy”.

Additionally, we sought to test whether the stimuli used for our first trustworthiness manipulation were fit for purpose as indicators of trustworthiness. Our trustworthiness items were information to be presented on a fact sheet, containing 15 questions about each character with multiple answers given as neutral facts or ones intended to frame the character as trustworthy or untrustworthy in a social context. Questions were the same for both characters. These questions included; “What did she do at University?”; “What does she do for a living?” “What do her colleagues say about her?” and “What did she do last weekend?”, as well as presenting an employer reference. For the 30 total sample statements, participants rated their trustworthiness on a scale from 0 to 100 (untrustworthy to trustworthy). Our full materials for Stimulus selection can be found on the Gorilla open repository, at https://app.gorilla.sc/openmaterials/668128.

## Results

Faces and voices that were rated most similarly for trustworthiness were chosen for the characters of ‘Anna’ and ‘Beth’ (respective ratings: Faces M = 51.67, SD = 13.11, M = 50.80, SD = 25.74; Voices M = 53.73, SD = 4.92, M = 52.75, SD = 6.14). This provided two pairs of stimuli which were in the middle range of trust ratings, hence being reasonably neutral and suitable to use for both trustworthy and untrustworthy conditions.

For our fact sheet items, 27 statements matched the modal response for trustworthiness based on their intended design (trustworthy statements were rated as trustworthy, neutral as neither trustworthy nor untrustworthy, untrustworthy as untrustworthy). The final three statements were removed or edited such that the number of statements was the same for both characters. For each character there were 11 final trust statements and two filler/neutral statements. One filler was the same for both characters (received a 2:1 at university and is still in contact with friends) where the other indicated for each character good competency/likeability in their respective jobs (being offered a graduate scheme by her employer and receiving good tips at work respectively). For the list of ratings for each statement, see data on OSF.

## Study 1

In Study 1, we aimed first to determine whether the Wayfinding Task was capable of reflecting trusting behaviors in our virtual characters. To this end, we employed a simple trustworthiness manipulation consisting of socially salient information (the ‘fact sheet’, outlined in *Design*). This effect of trustworthiness was hypothesized to be demonstrated in participants’ behavior during the Wayfinding Task; namely following advice, which character was asked for advice first (on trials where both characters were asked), which character was asked for advice more frequently overall, and the average interpersonal distance between the participant and each character on asking for advice. These dependent variables were maintained for all studies in the current paper.

Although there are considerations to be taken into account for remote HMD testing, mostly relating to recruitment rates [49], previous research has indicated that carrying out HMD-based research in home environments is feasible [50, 51]. Hence, we also sought to determine whether remote testing could yield similar success for the present work.

### Methods

#### Participants

A power analysis was conducted using G*Power [52], based on the second study of Hale et al. [24] which of Hale’s work most closely resembled our own. The effect size for “approaching for advice” in Hale’s study was d = 0.75. This analysis indicated a minimum yield of 20 participants would provide power of 0.8 to detect an effect of at least d = 0.75 at an α level of .0125. As ours was a new task, and to account for potential exclusions, we aimed to recruit more participants, resulting in a target sample of 36. We excluded participants from taking part if they had a history of psychiatric or psychological disorder, if they were under 18 years of age, if they indicated that they did not take the experiment seriously (see Post-test questionnaire) or if they did not complete the study. 71 participants were recruited, with 36 completing the full study and therefore subject to analysis. 3 of these 36 did not complete the requisite number of trials in the Wayfinding Task and therefore the remaining 33 were subject to analysis. Data were collected between February and April 2021. Participants were given instructions to pseudonymize their data. This procedure was the same for all subsequent studies (see Procedure). Due to the nature of online recruitment, researchers had no access to personally identifiable data during or after data collection.

For the purposes of analyzing the IAT, we utilized similar exclusion criteria to Van der Biest et al. [37] who also used the modified IAT to assess associations with trustworthiness. As such any individual trials slower than 10,000ms within a dataset were removed before analysis, and we disregarded IAT data from participants who scored incorrectly on their first attempts at >40% of the trials in one block (congruent/incongruent) for the purposes of calculating D scores.

In the final sample of 33 participants, ages ranged from 18–54 years (M = 29.49, SD = 10.40), 4 participants identified as female and 29 as male. Participants were recruited from social media, predominantly Reddit. Participants were compensated for their time via Amazon vouchers. All owned either an HTC Vive or Oculus Rift S with SteamVR. Participants were randomly assigned either character to be trustworthy. Overall, 15 were assigned to the ‘Anna trustworthy’ condition, and 18 to the ‘Beth trustworthy’ condition. Numbers in the different counterbalancing conditions were uneven due to the random nature of exclusions, drop-outs, and no-shows. Ethical approval for this study was granted by King’s College London’s Research Ethics Committee, registration number MRSU-20/21-21154. Ethical standards herein conform with the declaration of Helsinki, and participants provided informed written consent to take part.

#### Materials

*Apparatus*. Links to the study on Gorilla limited recruitment to computers using Chrome browsers, with no limitations on location or connection speed. Our application (the Wayfinding Task) was implemented in Unity 2019.4.8f and tested for use with the HTC Vive and Oculus Rift S. As the requirements for these (HTC Vive: Intel Core i5-4590 equivalent Processor, NVIDIA GeForce GTX 1060 equivalent GPU, 4GB RAM with HDMI 1.4 equivalent; Rift S: Intel i3-6100 equivalent Processor, NVIDIA GTX 1050 Ti equivalent Graphics card, 8GB RAM with Compatible DisplayPort) should be met by any computer running our Wayfinding Task, these exceeded the minimum software requirements. Code for our Wayfinding Task is available via our repository on Github: https://github.com/zcbtmfc/Wayfinding-Task.

#### Design

*Trustworthiness manipulation*. The studies in Hale et al. [24] which were conducted in immersive VR used an interview between characters and participants to manipulate trustworthiness. Despite its stated purpose of manipulating trustworthiness, the content of the interview in their study was not necessarily related to trustworthiness. For example, the statements “we like to get stuck in local culture, so we don’t really go to touristy places” and “lie in the sun and drink cocktails… that’s pretty much all I want to do” do not seem to manipulate trustworthiness but other facets of personality. While this may have been a strategy to not make the manipulation or study aim too obvious, it is also possible that these other facets of personality interact with the main manipulation. For example, likeability and warmth are highly correlated with trustworthiness for voices [48]. Hence, we focused our directing questions on social information related to others’ opinions about our characters’ trustworthiness and reliability. Our questions were selected based on the outcome of our prescreening (see Stimulus selection).

Participants were instructed to read through all of the materials and were shown the face and name of each character. Each question was presented on screen one at a time, with the character’s face in view. Both characters had contrasting answers relating to their trustworthiness. Throughout the answers, the trustworthy character was portrayed more favorably in a social context. For example, a trustworthy statement to the question “What do her colleagues say about her?” would be “I often confide in her, and she has never discussed my issues with others,” in contrast to the untrustworthy statement “I told her I had a weird rumour being spread about me. The next day I heard her spreading it further and discussing it with the other waiters and waitresses”. In Study 2 of Hale et al. [24] they also reported that interview order has a significant effect on ratings of rapport, which in turn potentially affected maze behavior. Hence the presentation order of trustworthy/untrustworthy was counterbalanced across participants, along with which character was rendered as trustworthy/untrustworthy. For full transcript of the fact sheet, see S1 File.

**Fig 1:**
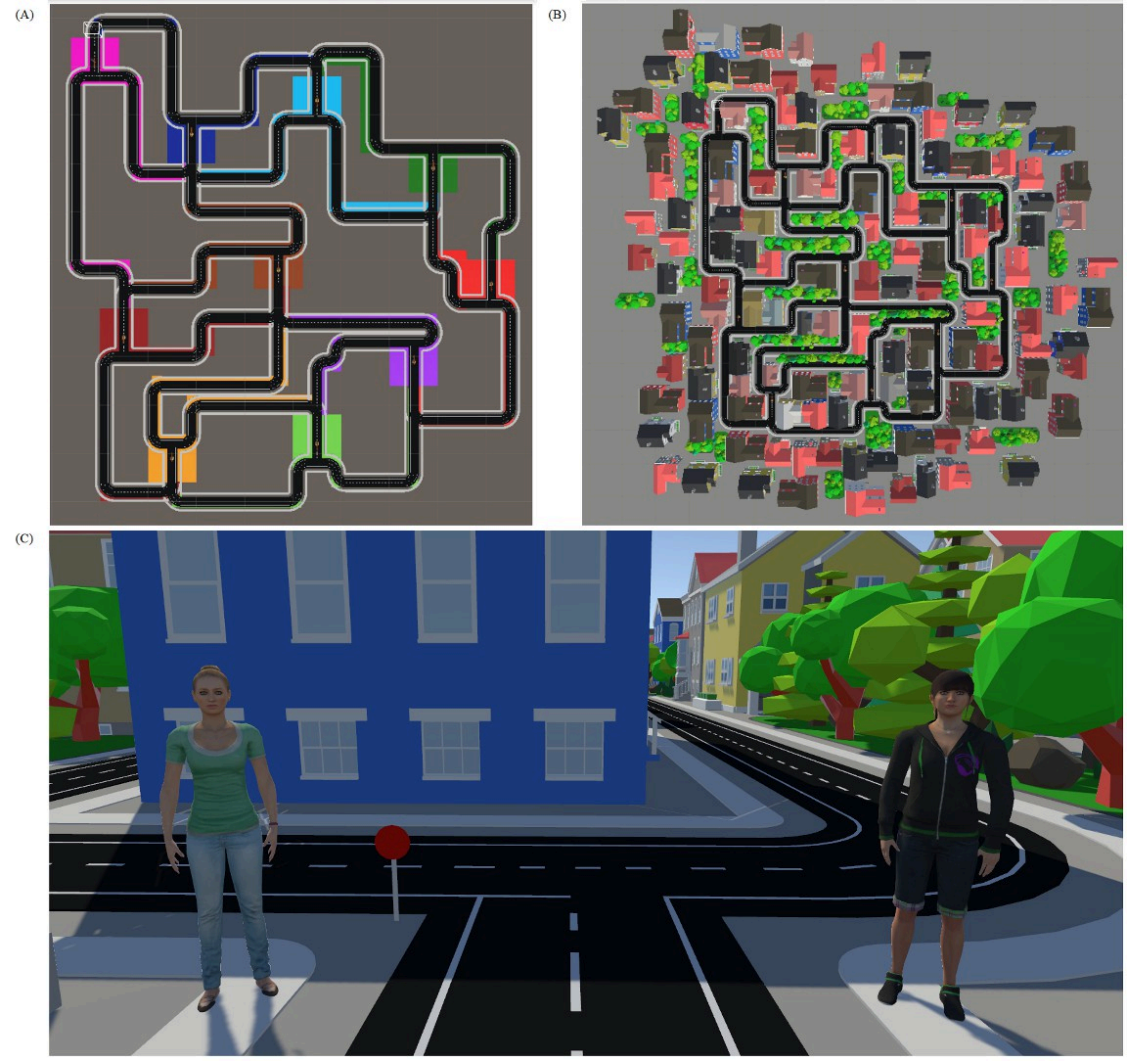
Views of the Wayfinding Task. (A) The shape of the layout of our Wayfinding Task. (B) Bird’s eye view of the Wayfinding scenery. (C) Beth (left) and Anna (right), positioned in a room of the Wayfinding Task just before left/right crossroads.

*Wayfinding Task*. In contrast to Hale et al. [24]’s design, which consisted of isolated chambers (trials) with two doors at the end of each chamber, and where each room was linked via a maze corridor, our Wayfinding Task was designed to be navigated with more agency. Each fork in the road allowed movement through one of the selected paths to the next fork in the road with any number of exploration patterns of the city map being possible, as participants could walk forward freely in any direction. This aimed to give a feeling of agency and continuity with the environment (Fig 1A and 1B). The two characters appeared as part of the environment, before each set of branching paths (Fig 1C) and could be interacted with to ask for advice. At any given crossroads, participants could ask one, both, or neither character for advice. The position of the character was randomized between the left and right, and the number of times each appeared on either side was counterbalanced within participants. Participants were only told how to consult the characters for advice, via a press of the trigger on their controllers; it was not explicitly instructed that they had to ask any combination of characters at any given time. ‘Asking’ a character in this manner would prompt the character to speak advice aloud. At each crossroads there were two possible paths to choose (left or right) and each character advised the participant to choose one of the two possible paths. Advice was given independent of the other character, so on 50% of trials their advice was the same. This served the purpose of reinforcing how the Wayfinding Task was not a manipulation of trust, but purely a measure, as participants could not infer that one character was giving ‘correct’ advice and could thus only infer trustworthiness based on the results of our prior manipulation. Additionally, by showing that advice was contradictory at points, but not continuously throughout, this provided an incentive for participants to ask both characters on some trials, and thus for us to measure who was asked first (see Dependent Variables below). The final reason for ensuring the two characters’ advice was given independently was that, if the two characters’ advice had differed on every trial, participants could develop a strategy of only ever approaching one character, knowing that the other character would give opposing advice. In principle therefore a participant could consistently approach the untrustworthy character and then always disregard their advice. In this situation we would not be able to determine whether the participant was truly following the trustworthy character’s advice.

All paths connected to new crossroads (Fig 1A), meaning there was no correct or incorrect decision. The task ended after 16 paths were chosen. Participants were instructed that “Your objective is to explore the maze.”. As the task was framed as a maze and designed to look reminiscent of an unfamiliar and complex urban environment, we would expect participants to request advice on exploration from the character who was more strongly associated with trust, regardless of not having a specific goal. The task was self-paced, and participants were advised to take a break if they were suffering adverse effects (see Post-test questionnaire for a full list of effects). Otherwise, the entire wayfinding procedure took place as one continuous session. Each character model was assigned one of the two voices, matched on trustworthiness from the stimulus selection, which they kept throughout.

*Dependent variables*. We calculated the interpersonal distance between participants and each character in virtual space on asking for advice; which character was asked first on each crossroad; the frequency with which each character was asked overall; and the frequency with which each character’s advice was followed. Interpersonal distance was computed as the average distance to each character per participant, at the point when the participant pressed the button on their controller to ask for advice. For example, if a participant were standing 0.5 meters away from a character when they had pressed the trigger to ask for advice, the interpersonal distance for that trial and character would be logged at 0.5m. In our program, these are logged as Unity units, which are equivalent to meters for the purposes of our studies. For which character was asked first, as participants could only ask one character first per trial, we calculated the percentage of trials in which each character was asked first (out of all 16 trials). Whether each character was asked for advice was calculated individually for each character was calculated as a number out of the 16 possible trials on which they could be asked. These values hence range from 0–16 for both of our characters, reported as frequency. Finally, we calculated our rate of advice following. Participants were determined to have followed advice only if they asked a character for advice and then traveled in the direction the character suggested. As there was a possible overlap for both characters (both gave the same advice on 50% of trials), this was again computed individually for each character. Thus, if a character was asked for advice and that advice was followed, this was scored as following that character’s advice, irrespective of whether the other character was also asked or not. This means that for each character the advice following frequency is a number out of 16 trials in which their advice was both sought and followed.

*Implicit Association Test*. The modified IAT was presented after the Wayfinding Task to provide an additional quantitative measure of trust in conjunction with our Wayfinding Task, by assessing whether either character was more implicitly associated with trust [38]. We positioned this after the Wayfinding Task to avoid priming participants on the term of ‘trust’. This paradigm consisted of five blocks. Throughout all blocks, participants had to press one of two keys which related to an attribute displayed in the top corners of the screen. If they got the answer incorrect, a red ‘x’ would appear on the screen and they would not be able to proceed until pressing the correct button. Block 1 had the attributes ‘Anna’ and ‘Beth’. The faces of each character would appear in the center of the screen, and participants had to match the faces to their respective names. This procedure was completed for 12 trials. Block 2 had the attributes ‘Trustworthy’ and ‘Untrustworthy’. Participants would press these buttons as terms appeared on screen. These terms included reliable, honest, loyal, responsible, honourable, truthful and dependable, as well as their antonyms; and were selected based on use in a previous trust IAT [53] and a study investigating the determinants of trust [54]. This procedure was completed for 14 trials. Block 3 had the attributes ‘[Trustworthy Character] or Trustworthy’ and ‘[Untrustworthy Character] or Untrustworthy’, where [Character] boxes were either of the two character names. As the trustworthy character shared the label of ‘trustworthy’ for our button presses and vice-versa, this was the ‘congruent’ condition. In the center of the screen would appear a character face (Anna or Beth) or an attribute, for 26 trials. Block 4 had the attributes ‘Trustworthy’ and ‘Untrustworthy’, in reversed positions from Block 2 (so using the opposite buttons). Other than this, the procedure was the same as Block 2. Block 5 had the attributes ‘[Untrustworthy Character] or Trustworthy’ and ‘[Trustworthy Character] or Untrustworthy’, where [Character] boxes were the character names. As the trustworthy character shared the label of ‘untrustworthy’ for the associated button presses and vice-versa, this was our ‘incongruent’ condition. As in Block 3, in the center of the screen would appear a character face (Anna or Beth) or an attribute, for 14 trials. For the purposes of counterbalancing, we paired Blocks 2 and 3 (the ‘congruent pair’) and Blocks 4 and 5 (the ‘incongruent pair’). This would mean the order of Blocks was either 1 -> 2 -> 3 -> 4 -> 5 or 1 -> 4 -> 5 -> 2 -> 3; with participants completing either the Congruent or Incongruent trials first, respectively. Each pair was assigned based on the position of attributes, as 2 and 3 had the Trustworthy attributes in the top left, and 4 and 5 had Untrustworthy in that position.

Response times on each trial were measured from onset of stimulus until button press. The variable of interest used to calculate D scores was the difference in mean response time between Congruent and Incongruent trials (blocks 3 and 5). The results of the IAT would hence indicate whether participants had maintained an association between our characters and trust/distrust after the manipulation.

*Post-test questionnaire*. Finally, participants received some questions about their experience. In particular, we asked questions about their adverse responses to VR, including whether they experienced the following effects: motion sickness, queasiness, headaches, and eye strain. This was followed by a debrief including instructions on how to locate and upload the files from the wayfinding experiment into a Dropbox folder and a general Debrief, which outlined the aims of the study, how trustworthiness was manipulated in each character and our dependent variables, as well as a brief summary of the IAT and our questionnaire. We also asked “Did you participate seriously and attentively at all stages of the experiment (reading the factsheet, VR Wayfinding Task, reaction time task, post-test questionnaire)?”.

#### Procedure

**Fig 2:**
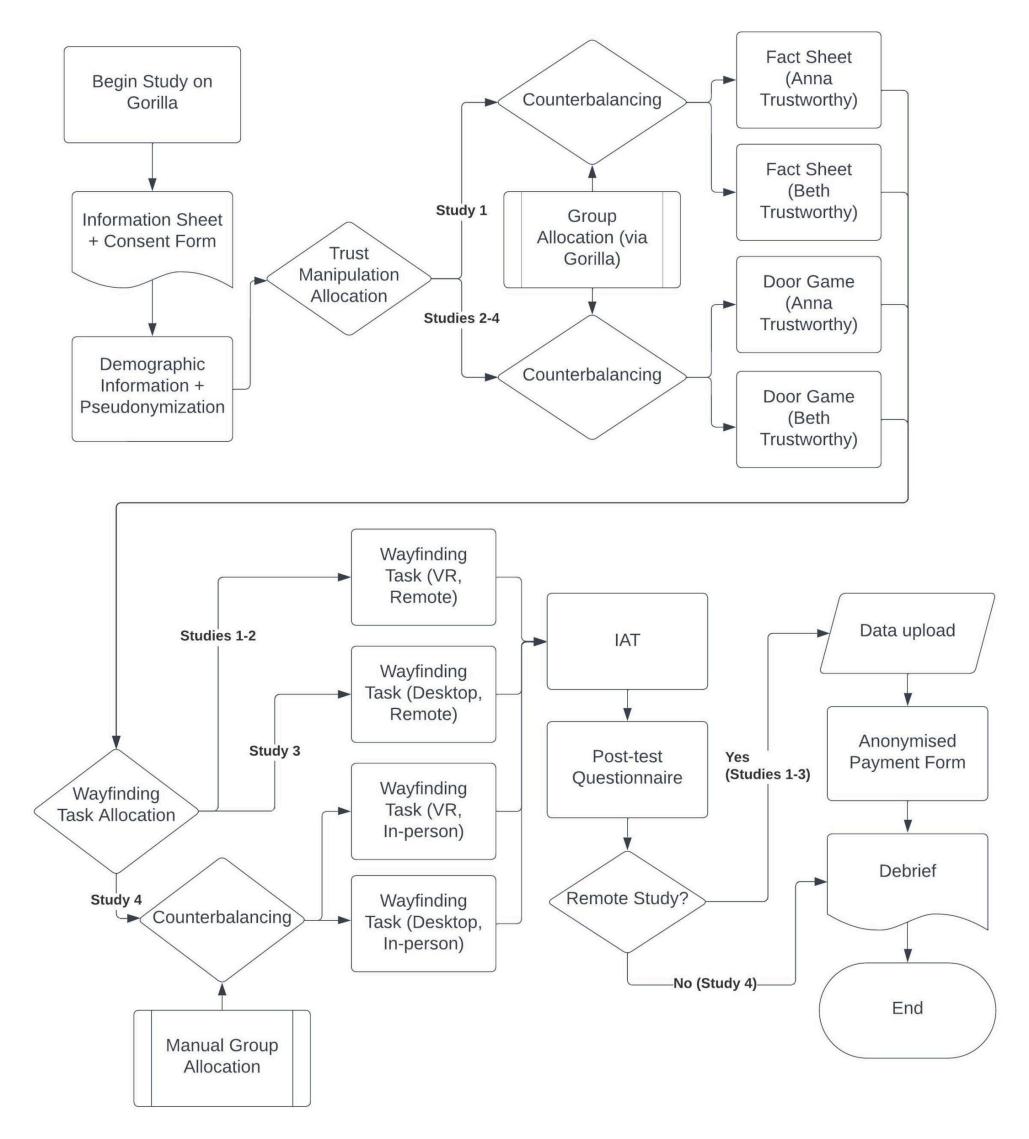
In-study procedure.

Advertisements on social media included institutional affiliations, a brief outline detailing which tasks were to be completed, notice of compensation and a recruitment email which prospective participants should contact, confirming that they did not meet exclusion criteria. On responding and fulfilling our recruitment criteria, participants were sent materials to complete the VR part of the study, as well as a more in-depth outline and instructions to contact the email again if encountering technical difficulties, as well as expected response times from the researchers. Materials included the.exe file running the Wayfinding Task, as well as instruction to launch the file in SteamVR at the time indicated by the experiment (see below). Participants were also informed that they could test the program before running the experiment to ensure compatibility with their software and headset. Participants were presented a link to the Gorilla Experiment Builder (hosted at www.gorilla.sc). Upon accessing this link, they could click a button to begin the study, where they would be presented with an Information Sheet explaining that they were taking part in research on decision-making in a virtual environment. This also reiterated the exclusion criteria, hardware and software requirements, as well as outlining the study and potential risks and data handling, as compliant with our ethical clearance (see Participants). They then signed a consent form, entered their age and gender and went through instructions to generate a pseudonymized code for data handling purposes, before proceeding to our trustworthiness manipulation and then a placeholder screen telling them to launch the Wayfinding Task (for full breakdown of the in-study procedures, and how they differ between each of the studies presented in this paper, see Fig 2). Finally, participants were instructed to return to Gorilla to complete our IAT and post-test questionnaire before receiving a link to submit an email for payment, and finally proceeding to the Debrief, outlining our dependent variables and the purpose of our questionnaire in more detail. Gorilla materials are available at https://app.gorilla.sc/openmaterials/560189.

The order of tasks for participants, and where allocation to versions of tasks diverged across each study.

### Results

#### Wayfinding Task

**Fig 3:**
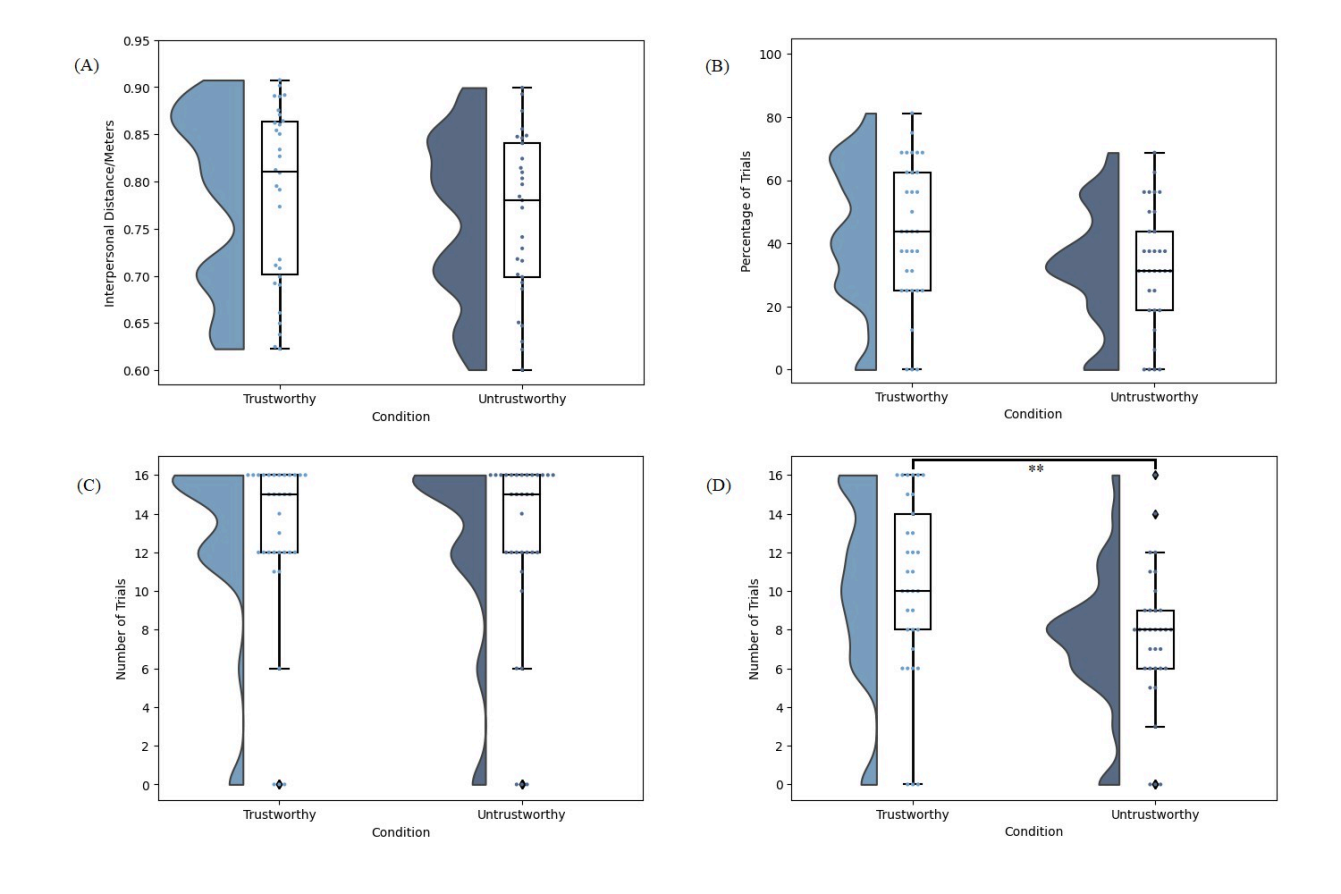
Study 1 data. (A) Distribution of the mean interpersonal distance per participant between the participant and each character on asking for advice. (B) Distribution of percentage of trials per participant on which a given character was asked for advice first. (C) Distribution of the frequency at which each character was asked for advice overall. (D) Distribution of the frequency at which each character’s advice was followed. For all panes, the responses of individual participants are represented by dots and bars indicate standard error. ** p < .01.

For all studies herein, data were tested for normality and the relevant nonparametric test applied where indicated. Statistical analysis was run using JASP Version 0.16.4.0 [55]. For t-tests, our test of normality was the Shapiro-Wilk test. We corrected for multiple comparisons in the Wayfinding Task, where we had four dependent variables, by adjusting our α value to .0125. Data for all four dependent variables in Study 1 are presented in Fig 3. All frequencies are out of a maximum possible total of 16 trials. Due to issues data logging in other versions of Excel, we excluded three participants for the interpersonal distance variable. As some participants chose not to ask certain characters at all, this also resulted in null values for certain conditions which affected our degrees of freedom (see OSF data).

For each participant, the mean interpersonal distance between the participant and each of the characters on asking for advice was calculated (see Fig 3A). A paired samples t test comparing the interpersonal distances between the participant and the trustworthy (M = 0.78m, SD = 0.09) vs untrustworthy character (M = 0.76m, SD = 0.09) trended towards a greater distance to the trustworthy character, but this did not survive our α correction, t(26) = 2.31, p = .029, d = 0.44.

A paired samples t-test indicated that the percentage of trials in which the trustworthy character was asked for advice first (M = 40.34, SD = 23.91) trended towards being higher than the percentage of trials in which the untrustworthy character was asked for advice first (M = 29.92, SD = 20.72), but this did not survive our α correction, t(32) = 2.18, p = .019, d = 0.38. A Wilcoxon signed rank test indicated that the frequency out of 16 trials at which the trustworthy character was asked for advice for advice overall (M = 11.70, SD = 5.43) was not significantly higher than the frequency at which the untrustworthy character was asked for advice (M = 11.61, SD = 5.60), W = 8.00, p = .500, r = 0.07.Finally, a Wilcoxon signed rank test indicated that the frequency out of 16 trials at which participants followed the trustworthy character’s advice (M = 9.58, SD = 5.21) was higher than the frequency at which participants followed the untrustworthy character’s advice (M = 6.91, SD = 3.84), W = 237.00, p = .003, r = .72.

#### Implicit Association Test

We calculated D scores for the IAT according to the standard protocol outlined in Greenwald, Nosek and Banaji [38]. A positive D score indicates a faster time on the congruent than the incongruent task. One participant was excluded as they answered incorrectly on their first attempt on over 40% of trials within a block.

A one sample t-test indicated that D scores (M = 0.51 SD = 0.43) were significantly greater than 0, t(31) = 6.73, *p* < .001, d = 1.19. This indicates that participants were faster at the congruent than the incongruent task, suggesting that our trustworthiness manipulation was successful and maintained to the end of the study.

#### Post-test questionnaire

Four participants (10.81% of the sample) reported adverse effects. Of these, three reported suffering from motion sickness as some point during the experiment, and one from eye strain.

### Discussion

We aimed to implement a new version of the virtual Wayfinding Task and to test whether it was sensitive to differences in trustworthiness of two characters, manipulated via social information provided in a fact sheet. In line with the findings of Hale et al. [24], we observed effects of trustworthiness on following the character’s advice, and a trend towards asking for advice first. We also observed a trend towards an effect for interpersonal distance, where the trustworthy character had a greater distance from the participant. The IAT data further verify that the fact sheet worked as a manipulation of trustworthiness, and that the effect of this manipulation was maintained until the end of the study.

Our remote study showed a high rate of attrition. We postulate that this could be due to numerous factors, such as disinterest, lack of motivation to continue the study, technical difficulties at different stages of the procedure, or other difficulties associated with lack of supervision. However, as these rates are similar to other remote studies [49], we do not believe that these reflect in any particular fashion on the results presented. As our attrition rates vary predictably across study design and we later complete an in-person study with a larger sample (Study 4), we will reflect on this trend between our remote studies and in comparison to our in-person study in the General discussion.

We also do not believe our adverse effect rate (10.81%) would unduly affect our results. As this was reported at the end of the experiment (and participants were aware of their right to withdraw), the sensation was not too uncomfortable to impede progress and since trustworthiness was manipulated within-participant, any negative sensations should affect judgements of both characters equally. Additionally, as this group was unsupervised, participants were free to self-pace and proceed as comfortably as possible; a condition which we maintained throughout subsequent unsupervised (Studies 2 and 3) and supervised study (Study 4).

We observed a trend towards an effect on interpersonal distance. Our findings indicated a greater distance between the participant and the trustworthy character compared to the untrustworthy character on asking for advice. This is in line with findings in perspective distortion, which demonstrate that a distance within a participants’ personal space correlates with lower investments in a trust game, and lower ratings of trustworthiness, as opposed to judgments made outside of this space [36]. Thus, increased distance may be an attempt to discern the features of a trustworthy character more clearly by positioning outside of this space, while no attempt would be made if already perceived as trustworthy. However, there are limitations to the interpretability of this finding. For one, while this previous work did control for facial expression, size, and lighting, as all accounted for in our Wayfinding Task, it was presented within an ongoing trust manipulation. However, this was also the case for work showing the opposite effect, which was performed with confederates [35]. Our implementation of the Wayfinding Task also limited the maximum interaction distance to a little over a meter, which may not be outside all participants’ personal bubble (the physical dimensions of space in which they are comfortable interacting with others). As a method of determining whether this was the case, and for contextualizing our findings for interpersonal distance with further studies, from Study 2 onwards we added a question asking participants to estimate the size of their ‘personal bubble’ (see General discussion for follow up). We consider that in future it may be useful to replicate such a setup with more lax parameters for interaction.

It is interesting to note that, while we did not see a significant effect on either of the ask measures in our study, we noted a strong effect on following, and a trend towards an effect in terms of asking first. This effect and the IAT both provide evidence that our fact sheet was successful at manipulating participants’ levels of trust, and that this was reflected in the Wayfinding Task. Use of the fact sheet is in line with previous work indicating that access to social information is a strong predictor of trust [21]. In contrast, previous ask-endorse studies have used the accuracy of characters’ statements to manipulate trustworthiness [33]. Our success here may therefore indicate that this perceived accuracy is not a core component of trustworthiness manipulation during the ask-endorse paradigm, but instead is a dimension of trust, perhaps similar to predictability [31], which is sufficient in establishing trusting behaviors. However, as the effect of the trustworthiness manipulation on our outcomes was more limited than expected, it is also worth observing how the dependent variables continue to be affected by subsequent studies, and so we will observe and comment in respect to trends in the data as they develop. However, to maintain consistency with prior work using perceived accuracy to manipulate trustworthiness, our subsequent studies used a different manipulation to further validate the Wayfinding Task.

## Study 2

In Study 2, we sought to explore our behavioral effects in the Wayfinding Task with a new trust manipulation; the Door Game (again outlined in *Design*). While the fact sheet was successful at inducing trustworthiness, the presentation of a written document to introduce one to a stranger may be of limited ecological validity when compared to the ‘person on the street’ design of our Wayfinding Task. Additionally, the fact sheet does not provide any behavioral feedback as to whether one’s belief about a given character seems consistent with their behavior [56]. As there was no way to confirm the accuracy of the claims being made about our characters beyond hearing them from different people, or to personally compare the claims to their behavior, the fact sheet manipulation may also be susceptible to individual differences in generalized trust. Therefore, we decided to introduce a new manipulation which incorporates some behavioral feedback, while continuing to avoid the participant needing to input monetary-based value judgements as in investment games.

Through giving feedback regarding the outcome of our characters’ advice, we hoped that participants could infer their accuracy in a similar way to the behavioral manipulations used by Koenig and colleagues for ask-endorse [33, 57–60]; and that we could effectively influence trusting behavior in the Wayfinding Task in a population of adults using this behavioral manipulation. This may then demonstrate more explicitly that our methodology is in line with previous versions of the ask-endorse paradigm.

### Methods

#### Participants

A power analysis was conducted using G*Power based on our principal finding from Study 1 (the rate of following trustworthy characters’ advice, r = .717) which indicated a minimum sample size of 22 would be required to detect an effect of at least this size at power 0.8 and α level .0125. We excluded any participants who took part in Study 1 and used the same exclusion criteria otherwise. 68 participants were recruited. 32 completed the full study; 2 of these remaining 32 did not complete the requisite number of trials in the Wayfinding Task and therefore the remaining 30 were subject to analysis. Data were collected during September 2021.

In the final sample of 30 participants, ages ranged from 18–47 years (M = 29.1, SD = 8.97), 3 identified as female, 26 as male, and 1 as gender diverse. Participants were recruited via posts on Reddit and compensated for their time with Amazon vouchers. Participants could apply with any headset compatible with SteamVR. 16 participants were assigned to the ‘Anna trustworthy’ condition, and 14 to the ‘Beth trustworthy’ condition. Ethical approval for this study was granted by King’s College London’s Research Ethics Committee, registration number MRSP-20/21-25585.

#### Design

**Fig 4:**
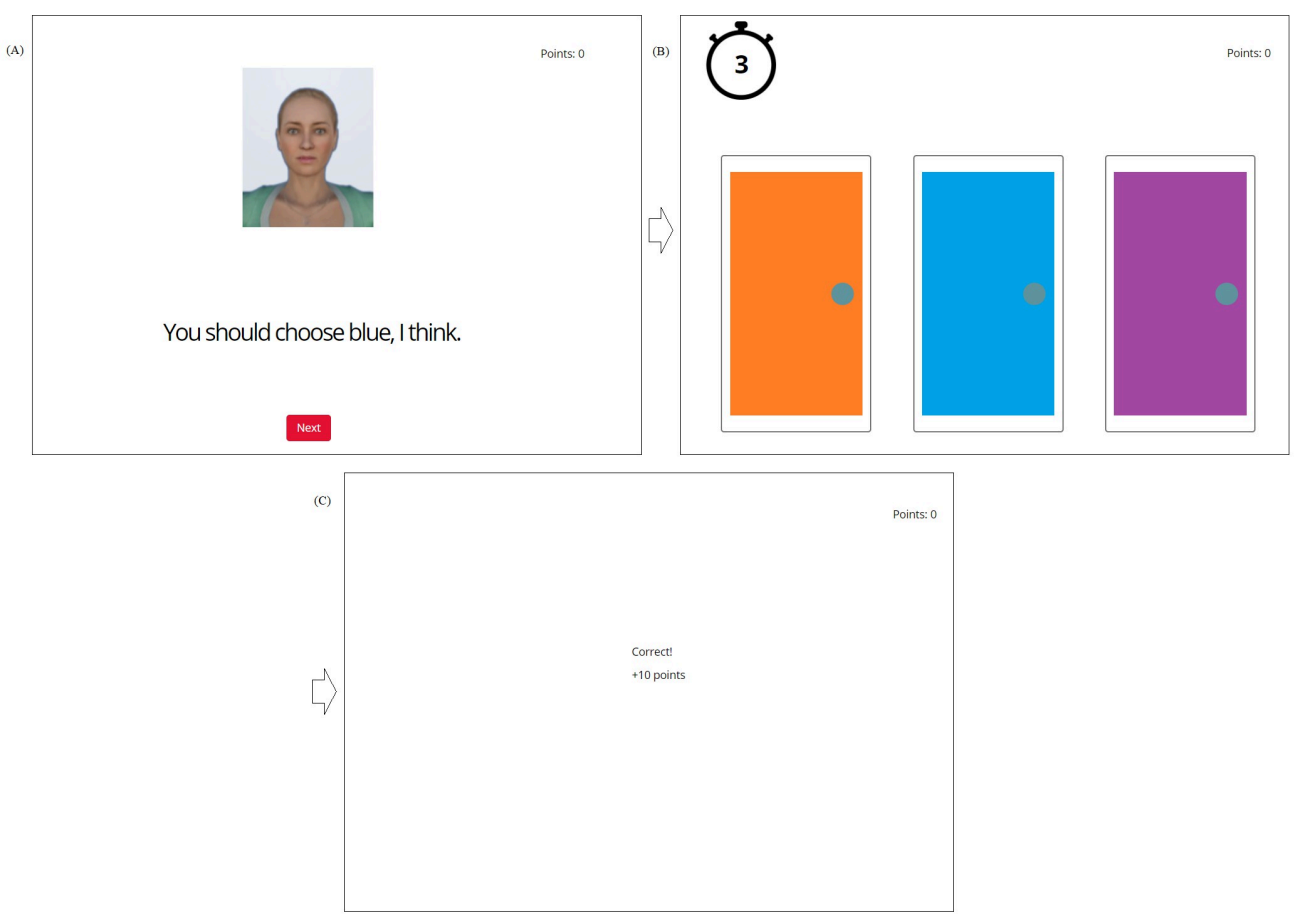
Structure of the Door Game. (A) Advice screen. (B) Door selection, including timer. (C) Feedback, after which the score in the top-right updates. Screen borders and arrows are for illustrative purposes only.

*Trustworthiness manipulation*. Our new trustworthiness manipulation, the Door Game, was structured to mimic that of Van der Biest et al. [37]. Participants were instructed to maximize their points total by selecting the correct door out of three, with help from our characters. Each door would either be correct (+10 points), incorrect (-10 points), or neutral (±0 points). Participants were introduced to our two characters, Anna and Beth, by name and picture; and told that one of the two characters would offer them advice before each set of doors, which they would see for about 5 seconds and may choose whether or not to follow (for introduction script, see S2 File). For example, for the ‘advice’ screen, participants may see an image of Beth saying “You should choose blue, I think” (referring to the blue door, see Fig 4). They were told they would then have about 5 seconds to choose one of the doors before receiving feedback. Each sequence of these three screens (advice, doors, and feedback) counted as one trial, for 36 trials total, as in the original design [37]. Advice screens alternated between characters on each trial. The trustworthy character would always indicate, by color, the correct door, while the untrustworthy character had a 1 in 3 chance of indicating the correct, incorrect, or neutral door. Each color door had an equal chance of being correct, incorrect, or neutral for any given trial. Each color door stayed in the same position, while the number of these outcomes was counterbalanced. As such, the aim was for participants, over the course of the Door Game, to associate one character with trust in their advice. As in Study 1, we verified whether these associations existed, and if they were maintained to the end of the study, through use of the IAT. Points did not correspond to any real-world incentives, for example monetary value. Our Door Game was constructed natively in the Gorilla Experiment Builder, which continued to host our study (gorilla.sc).

Our dependent variables concerning the Door Game reflected both our IAT and Wayfinding Task variables. These consisted of two comparisons; reaction times concerning the trustworthy vs untrustworthy character, where shorter reaction times are likely to indicate a greater certainty in one’s response, consistent with trusting the character’s advice; and the number of times each character’s advice was followed out of their 18 trials, which we hypothesize will be greater for the trustworthy character as each participant learns that their advice yield greater points.

*Wayfinding Task*. From this study onwards, we integrated gestures to the responses of each character. They would sync their mouths with speech and gesture their arms in the direction that they advise. This was done as an attempt to increase realism further. All other aspects of the Wayfinding Task were the same as for Study 1.

*Post-test questionnaire*. Questions from Study 1 were also included in this study. From this study onwards, we also added the question ‘how big do you estimate your personal bubble to be? (the gap you leave between you and another person when talking to them)’. This was an attempt to examine whether the parameters of the task for interpersonal distance were suitable. As this change was implemented partway through recruitment, we did not survey the full group. Additionally, while we asked for a size estimate, not all of these remaining respondents gave quantifiable answers. Of 21 respondents, 15 gave numerical units. For participants that gave a range of sizes for their bubble (for example, 1–2 meters), we took the average size (to use the prior example, 1.5 meters).

#### Procedure

Procedure was the same as in Study 1, with the Door Game taking the place of the fact sheet during the trustworthiness manipulation phase (see Table 1 and Fig 2). The IAT was implemented in the same manner as in Study 1. Gorilla materials are available at https://app.gorilla.sc/openmaterials/560208.

### Results

#### Door Game

For this and all subsequent studies, we corrected for multiple comparisons in the Door Game, where we had two dependent variables, through adjusting our α value to .025.

A paired samples t-test indicated that participants’ mean reaction times when receiving advice from the trustworthy characters were significantly lower (M = 835.93ms, SD = 340.35) than when receiving advice from the untrustworthy character (M = 1111.46ms, SD = 336.45), t(29) = 4.77, *p* < .001, d = 0.871.

A paired samples t-test indicated that the frequency out of 18 trials that participants followed the trustworthy characters’ advice (M = 16.70, SD = 2.10) was significantly higher than the frequency at which they followed the untrustworthy characters’ advice (M = 8.67, SD = 4.69), t(29) = 7.69, *p* < .001, d = 1.40.

#### Wayfinding Task

**Fig 5:**
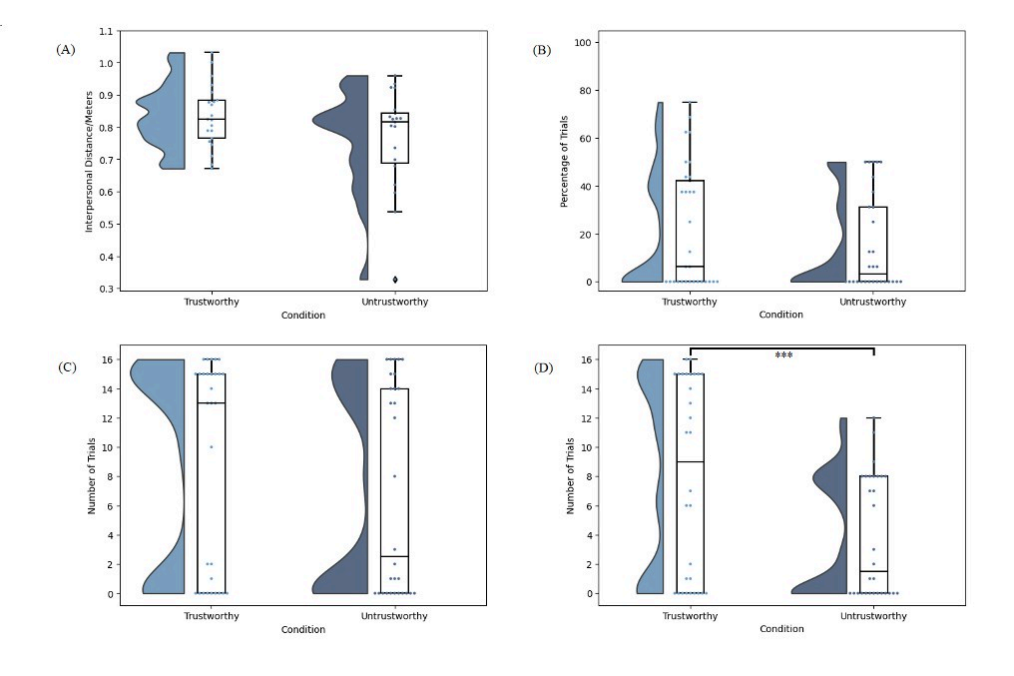
Study 2 data. (A) Distribution of the mean interpersonal distance per participant between the participants and each character on asking for advice. (B) Distribution of the percentage of trials per participant on which a given character was asked for advice first. (C) Distribution of the frequency at which each character was asked for advice overall. (D) Distribution of the frequency at which each character’s advice was followed. For all panes, the responses of individual participants are represented by dots. Bars indicate standard error. *** *p* < .001.

Fig 5 presents the data from each of the dependent variables in the Wayfinding Task for Study 2.

A Wilcoxon signed rank test comparing the interpersonal distance between the participant and the trustworthy (M = 0.84m, SD = 0.10) vs untrustworthy character (M = 0.76m, SD = 0.16) indicated that the difference was not significant, W = 120.00, *p* = .142, r = .40.

A Wilcoxon signed rank test indicated that the percentage of trials in which the trustworthy character was asked for advice first (M = 21.88, SD = 25.68) was not significantly higher than the percentage of trials in which the untrustworthy character was asked for advice first (M = 15.42, SD = 20.08), W = 106.00, *p* = .083, r = .39, although the trend was in the same direction as in Study 1.

A Wilcoxon signed rank test indicated that the frequency out of 16 trials at which the trustworthy character was asked for advice overall (M = 8.93, SD = 7.19) trended towards being higher than the frequency at which the untrustworthy character was asked for advice (M = 6.87, SD = 7.11), but this did not survive our α correction, W = 96.00, *p* = .021, r = .60.

Finally, a paired samples t-test indicated that the frequency out of 16 trials at which participants followed the trustworthy character’s advice (M = 7.87, SD = 6.78) was higher than the frequency at which participants followed the untrustworthy character’s advice (M = 3.83, SD = 4.16), t(29) = 3.62, *p* < .001, d = 0.66.

#### Implicit Association Task

A one-sample t-test showed that D scores (M = 0.47, SD = 0.51) were significantly greater than 0, t(29) = 5.05, *p* < .001, d = 0.92. This indicates that participants were faster at the congruent task, suggesting that our trustworthiness manipulation was successful.

#### Post-test questionnaire

In terms of adverse effects, two participants reported motion sickness and one reported feeling queasy. This is an adverse effect rate of 10%. Of the 15 participants who responded with numerical data regarding the size of their personal bubble, the mean estimated size was 89.67cm (SD 53.57). Other responses included ‘medium’, ‘big’, or variations thereupon.

### Discussion

For Study 2 onwards, we aimed to implement a more implicit trustworthiness manipulation via the Door Game. Our implementation of the door game was shown to be successful in producing positive results in select outcome measures and the IAT, corroborating Van der Biest et al. [37]’s use of the Door Game to manipulate trustworthiness. We observed an effect of trustworthiness on following advice (corroborating our first study) and a trend towards an effect on the frequency of approach and which character was asked for advice first. However, there was no effect on interpersonal distance.

Our adverse effect rate was similar to that of Study 1. Given that none of our participants ended the experiment as a result, we argue the effect of these rates is negligible.

Regarding methodology and recruitment, Study 2 was open to more devices than those which the Wayfinding Task was natively developed on (the Oculus Rift S and HTC Vive), which merits discussion. Here, our aim was to expand our recruiting pool, which successfully hastened recruitment; from February-April in Study 1, to just September in Study 2. While no participants reached out to the researchers for technical advice in implementing the Wayfinding Task in VR, this may be an artefact of remote study making it take more time to troubleshoot, so they may have not felt this was worth it. Only two participants dropped out of the study at the stage of the Wayfinding Task, which is indicative of a low level of attrition due to technical difficulties. The only participant to report technical issues with an unspecified Pimax device also described the step they took to fix it in their device’s settings after the conclusion of the study. This is likely in part due to the demographic of recruitment, as a lot of owners of HMDs are likely more familiar with their settings and custom or developer software. We therefore take this as indicative of our program’s compatibility across devices.

Regarding the interpersonal distance, we observe a lack of a trend in Study 2. As we have discussed conflicting hypotheses regarding interpersonal distance, the lack of significant effects may be in part due to individual differences regarding distance and trust. However, in comparison to Study 1, we posit that this may be due to familiarity in design between the Door Game and the Wayfinding Task. As the decision making behavior is conserved between the Door Game and the Wayfinding Task (i.e. participants follow the trustworthy characters’ advice more often in both, once the relationship is learned), then participants may disregard interpersonal distance as it was not relevant during their initial learning period in the Door Game. We will continue to monitor and comment on trends throughout our proceeding studies with the Door Game in the General discussion.

Of our three other dependent variables, two concern the ‘ask’ portion of the ask-endorse (who was approached first, and who was approached more overall); and one concerns the ‘endorse’(whose advice was followed), while our novel outcome measure assesses how trust was expressed physically during interaction. In this study we found effects on the endorsement variable, and a trend which did not survive correction for multiple comparisons on both of the ask variables. It may therefore be informative to first compare the effects common to Hale et al. [24]. In Hale et al.’s paper, Studies 1 and 2 involved approaches in physical space, with the participant engaging via a projector-based display and HMD respectively. Both found significant results on all three ask-endorse measures, though these differed in the magnitude of results. For approaching first, these effects were d = 0.89 vs 0.97 for Hale’s Studies 1 and 2; similarly for approach overall these were 1.01 and 0.99 and finally 1.63 and 2.06 for following advice. This is also consistent with how HMD-based VR shows stronger immersion effects than other technologies [61], which may lead to more reliable results. In their third study, participants didn’t move and could only consult characters via phone call as they were not embodied in the environment. It is potentially this lack of immersion which explains why they only found marginally significant effects for following advice at 0.41, and no significance on other measures. For this reason, and the effect sizes shown above for earlier measures, we consider following advice to be the principal measure of trust in the Wayfinding Task. This is in line with our findings, where this variable showed greatest effect in terms of magnitude of effect size.

The trend of lower effects in Hale et al. [24]’s Study 3 compared to their earlier work continued for first approach and overall approach, at 0.29 and 0.58 respectively. As Hale et al. attribute these weaker effects in part due to their use of investment games as a manipulation, we also may consider contextualizing these weaker results concerning ask variables with how the Door Game developed from our fact sheet. While our frequency of trials was the measure which trended towards an effect, there was no effect on this measure in our previous study. However, the presence of a trend on one of these measures does indicate that there may be some effect. As the Door Game requires trust to be determined by first-hand behavioral inferences, we may posit that trust in this task is presented more ambiguously compared to the fact sheet and its presentation as a factual recollection of events. This would reflect the reduction of effect in Hale’s Study 3 where they use the investment game as a manipulation instead of factual interviews [24]. This ambiguity may lead to a similar number of requests for advice across both characters in our Study 2. This change could also be in part due to asking the trustworthy character for advice as a reference point against the other character. By presenting the same response on 50% of trials, which again was necessary to prevent further inferences on trust, this could have meant more trials spent ‘testing’ responses from the untrustworthy character. That no further inferences on trust were ultimately made from the Wayfinding Task is reflected by how participants did overall endorse the trustworthy character and continue to do so through further studies; though we will discuss this further in the General discussion as we observe overall trends across our studies.

Finally, the other component to which Hale et al. attributed their weaker effects was the non-immersive setup of their third study. Thus, it would be useful to determine whether the effect of trustworthiness on behavior in the Wayfinding Task is replicable in a non-immersive setting when keeping our trust manipulation constant. Hence, for our Study 3, we sought to examine whether our effects would persist in a non-immersive setup.

## Study 3

In Study 3, we employed a desktop setup (mouse-and-keyboard controlled, with display on the native monitor) of the Wayfinding Task, once again administered remotely in a self-supervisory context. This was the same Unity application, which therefore functioned the same as in our immersive VR condition, but with participants required to use the keyboard and mouse instead of respective controllers. Our aim here was to investigate whether the effect of our Wayfinding Task to measure trustworthiness was dependent on the higher immersion and higher acceptability which characterizes the experience of immersive, HMD-based VR equipment or if the task remained suitable for use in a desktop environment.

### Methods

#### Participants

A power analysis was conducted using GPower based on our principal finding from Study 2 (the rate of following trustworthy characters’ advice, d = 0.66) which indicated a minimum yield of 25 participants was necessary to provide a power of 0.8 to detect this effect at α = .0125. Data were collected between January and May 2022.We excluded any participants who took part in previous studies and used the same exclusion criteria otherwise. 73 participants were recruited from institutional participant pools and participated remotely. 11 did not submit wayfinding data. Of the remaining 62, 31 did not complete the required number of trials in the Wayfinding Task and a final 1 was excluded as they indicated that they did not take participation seriously via the post-test questionnaire. Therefore, the remaining 30 participants were subject to analysis.

In the final sample of 30 participants, ages ranged from 18–42 (M = 21.20, SD = 4.25), 16 identified as female and 14 as male. Participants were compensated for their time via Amazon vouchers. 16 participants were assigned to the ‘Anna trustworthy’ condition, and 14 to the ‘Beth trustworthy’ condition. Ethical approval for this study was granted by King’s College London’s Research Ethics Committee, registration number MRSP-21/22-26991.

#### Procedure

Our Wayfinding Task was the same as in Study 2, but presented on the native display of the computer instead of in a separate HMD. For the post-test questionnaire, we did not ask participants about the same adverse effects from the other studies as we would not expect significant effects from a desktop setup as from a HMD [62]. However, we did leave participants the option to discuss if they were disturbed by external factors during the experiment. All other tasks and procedure were identical to Study 2. Gorilla materials are available at https://app.gorilla.sc/openmaterials/560224.

### Results

#### Door Game

A paired samples t-test indicated that participants’ mean reaction times when receiving advice from the trustworthy characters were significantly lower (M = 986.42ms, SD = 200.40) than when receiving advice from the untrustworthy character (M = 1059.44ms, SD = 220.72), t(29) = 2.35, *p* = .013, d = 0.43.

A paired samples t-test indicated that the frequency out of 18 trials that participants followed the trustworthy characters’ advice (M = 16.83, SD = 1.46) was significantly higher than the frequency at which participants followed the untrustworthy characters’ advice (M = 11.33, SD = 4.89), t(29) = 6.07, *p* < .001, d = 1.11.

#### Wayfinding Task

**Fig 6:**
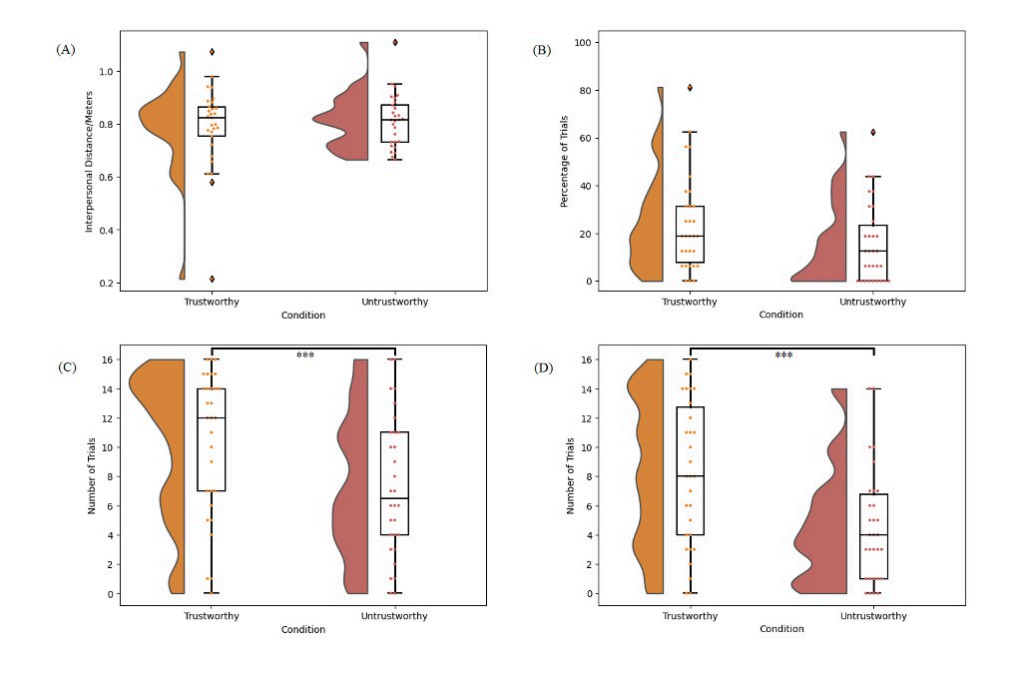
Study 3 data. (A) Distribution of the mean interpersonal distance per participant between the participant and each characters on asking for advice. (B) Distribution of the percentage of trials per participant on which a given character was asked for advice first. (C) Distribution of the frequency at which each character was asked for advice overall. (D) Distribution of the frequency at which each character’s advice was followed. For all panes, the responses of individual participants are represented by dots. Bars indicate standard error. *** *p* < .001.

Fig 6 presents the data for all dependent variables from the Wayfinding Task in Study 3.

A Wilcoxon signed rank test comparing the interpersonal distance between the participant and the trustworthy (M = 0.79m, SD = 0.16) vs untrustworthy character (M = 0.81m, SD = 0.10) indicated that the difference was not significant, W = 185.00, *p* = .695, r = -.09.

A paired samples t-test indicated that the percentage of trials in which the trustworthy character was asked for advice first (M = 23.96, SD = 20.11) trended towards being higher than the percentage of trials in which the untrustworthy character was asked for advice first (M = 15.63, SD = 16.64), but this did not survive correction for our α value, t(29) = 1.85, *p* = .037, r = .34.

A paired samples t-test indicated that the frequency out of 16 trials at which the trustworthy character was asked for advice overall (M = 10.43, SD = 4.93) was higher than the frequency at which the untrustworthy character was asked for advice (M = 7.30, SD = 4.75), t(29) = 3.68, *p* < .001, r = .67.

Finally, a paired samples t-test indicated that the frequency out of 16 trials at which participants followed the trustworthy character’s advice (M = 8.23, SD = 4.97) was higher than the frequency at which participants followed the untrustworthy character’s advice (M = 4.57, SD = 3.89), t(29) = 3.50, *p* < .001, d = 0.64.

#### Implicit Association Test

A one-sample t-test showed that D scores (M = 0.01, SD = 0.52) were not significantly greater than 0, t(29) = 0.15, *p* = 0.442, d = 0.03. This indicates that participants were not faster at the congruent task. **Post-test questionnaire**

One participant reported that the motion during the experiment gave them a headache, providing a reported adverse effect rate of 3.33%. Of 29 numeric responses to the size of their personal bubble, the mean estimated size was 79.00cm (SD = 38.84). Only one person gave a non-numeric response regarding their personal bubble, which was ‘average’.

### Discussion

In Study 3, we observed an effect on who was asked for advice overall, and on following advice for the trustworthy character, with a trend towards an effect for asking first. While we observed effects on reaction times and following for the Door Game, we also observed no effect on interpersonal distance, and for the first time, no effects on the IAT.

We observed a high rate of exclusions due to insufficient datasets in this Study. As our instructions, presented via Gorilla, for how to run the Wayfinding Task were the same in this study as compared to the previous two, this have been due to factors surrounding the differences in our sample. For instance, it may be due to less familiarity with running novel programs in our recruitment demographic, which may have led to technical incompatibilities, or misinterpretations as to how the program was supposed to work or conclude, which were not addressed; again, due to lack of supervision. However, factoring in these losses, our overall attrition rate was similar to that of our previous studies; Studies 1 and 2 had completion rates of 53.52% and 45.45%, respectively, while Study 3 had a completion rate of 42.47%. Thus this is an expected rate of data loss due to remote study, which, given replication of our principal finding, did not affect our results. We shall further discuss attrition in remote study upon reflection on the attrition rates of our in-person study, Study 4.

While we did not directly ask about adverse effects in a similar manner to other studies, one participant did report suffering from headache during the experiment. While this is not precise data for comparison to the other studies in this paper, this pattern may still be indicative of a general trend; it has been observed that adverse effects relating to VR are more frequently reported using HMDs than compared to desktop setups [62]. Additionally, our estimate of the size of participants’ personal bubbles continues to be within expected ranges (see General discussion).

Here it is worth exploring the use of the IAT to corroborate our relationship between participants and characters. Being an implicit, proxy measure of trust, there exists the possibility that the outcome does not reflect participants’ real attitudes, which seems in this case supported by every other behavioral measure in the Wayfinding Task supporting a trusting relationship. The IAT is especially sensitive in our design owing to its positioning; participants take part in the IAT after the Wayfinding Task, which means there exists the possibility of interference between establishing trust (via the Door Game) and measuring this relationship (post Wayfinding Task). It is therefore important to consider these results in comparison to the Door Game, which does not suffer from this potential for interference effects. In this study, we observe via the Door Game both reaction time effects, and an effect of following advice which was also corroborated during the Wayfinding Task, which we take to mean an effect of trust was observed as this aligns with our principal measure of trust (see Study 2 discussion). Whatever effect is lost seems to be reserved to our reaction time measures in the IAT, and given the similarities in design across our studies, is likely attributable to demographic. As participants were recruited from institutional participant pools, they were self-selecting on the basis of involvement with psychology studies rather than on their frequenting of specialist forums and social media relating to VR (in contrast to Study 1 and 2’s participants). This might make them more sensitive to the demand characteristics inherent in the IAT, which might not be the case for the more behavioral tasks preceding the IAT. As our Door Game showed a response in terms of the number of times characters’ advice was followed, reflecting our principal measure of trust in the Wayfinding Task (see Study 2 Discussion), we suspect that our failure to find reaction time outcomes on the IAT are not indicative of a failure of our trust manipulation. However, given the negative outcome for this study in contrast with the results of our Wayfinding Task, we consider the Door Game the more reliable of our confirmatory measures regarding trust manipulation.

Overall, Study 3 suggests that it is possible to measure trusting behavior using a desktop version of the Wayfinding Task in a remote testing context. However, the high attrition rate makes it difficult to determine the generalizability of this result. It is also possible that our earlier results could be particular to the population tested (those who own their own VR headsets). In our final study, therefore, we compared desktop and VR implementations of the Wayfinding Task directly in the same population, using in-person testing.

## Study 4

In our final study, we examined and compared immersive, HMD-based VR and desktop implementations of our Wayfinding Task using in-person testing. Comparing desktop setups to HMD-based VR can address whether the immersion aspect of VR [39] is a core component of replicating realistic behavior in the context of this type of social experiment. HMDs show stronger effects when compared to desktop virtual experiences [63, 64]. The comparison between these implementations would be difficult to make across our previous studies as participants in the immersive VR group (Study 2) were required to own and operate their own HMD, which may indicate a higher level of experience with computer games or similar immersive experiences compared to the desktop group (Study 3). Correspondingly, it has been shown that prior experience with VR affects participants’ judgement on perceptual quality [65], and a stronger visual realism enhances realistic responses [39]. By replicating the VR implementation in a population which may be less experienced, we may additionally increase the generalizability of our findings. Finally, while the previous study showed that we can achieve results consistent with trust using a desktop setup, the remote recruitment method poses its own set of limitations which may limit its accessibility to researchers; notably, high attrition rates or low recruitment [49] (see also General discussion). Therefore, it is in the interests of those who wish to employ these methods to know if the effect of this desktop setup, too, is replicable in the lab.

## Methods

### Participants

As Studies 3 and 4 were designed and conducted in parallel, the initial power analysis for Study 4 was conducted using GPower again based on our principal finding from Study 2 (the rate of following trustworthy characters’ advice, d = 0.66) which indicated a minimum yield of 25 participants was necessary to provide a power of 0.8 to detect this effect at α = .0125. We sought to increase the sample size to account for new statistical analysis. Data were collected from January to February 2022. Pseudonymization was again used to protect dataset anonymity during and after data collection. For in-person data collection, participant information was collected by the recruiting platform (Sona Systems, https://www.sona-systems.com/) in line with procedures approved by the local ethics committee. We excluded any participants who took part in previous studies and used the same exclusion criteria otherwise. 70 participants were recruited. 1 was excluded as they indicated that they did not take participation seriously via the post-test questionnaire.

In the final sample of 69 participants, ages ranged from 18–42 years (M = 21.46, SD = 5.09), 52 identified as female and 17 as male. Participants were recruited from institutional participant pools and were compensated for their time via Amazon vouchers. In the Desktop group, 16 were assigned to the ‘Anna trustworthy’ condition, and 16 to the ‘Beth trustworthy’ condition. 24 participants in this condition identified as female and 8 as male, with ages ranging from 18–41 (M = 21.19, SD = 4.43). In the immersive VR group, 21 were assigned to the ‘Anna trustworthy’ condition, and 16 to the ‘Beth trustworthy’ condition. In this study, numbers in these conditions were rendered uneven for the reasons discussed above (see Study 1 Participants) and due to manual allocation to HMD/Desktop groups prior to Gorilla’s automatic counterbalancing of the trustworthy character. 25 participants in this condition identified as female and 9 as male, with ages ranging from 18–42 (M = 21.47, SD = 5.14). Ethical approval for the study was granted by King’s College London’s Research Ethics Committee, registration number LRU-20/21-21153, with modification MOD-21/22-21153.

*Procedure*. Gorilla materials are available at https://app.gorilla.sc/openmaterials/560241.

*Design*. Our Wayfinding Task and trustworthiness manipulation remained unaltered from Study 3 for our immersive VR group. For the desktop group, we used the same Wayfinding Task altered for desktop functionality. The task was presented on a 1920x1080p display using a Dell Precision Tower 7910, running an NVIDIA GeForce GTX 1080 graphics card. For the HMD group, we used an HTC Vive.

*Post-test questionnaire*. Questions from Study 3 were also included in Study 4. In Study 4, we asked participants how many times they had used a VR headset on average. Responses could be ‘never’, ‘1–2 times’, ‘1–2 times a year’, or ‘on a monthly basis’. For the question regarding the size of their personal bubble, for participants that gave a size estimate alongside some rationale explaining deviations in their estimate (for example, one participant who stated “maybe a meter, but not sure. def more during covid times”), we took the numerical response to be their response for the purpose of calculating average/standard deviations (which in the example the above would be taken as one meter). For those who gave a size estimate using non-standard units (for example, one participant who stated “arm’s length”), we took no numerical size estimate.

#### Procedure

Participants were told in the advertisements that they could be assigned to either an HMD or Desktop-based condition. Participants visited King’s College London Psychology testing labs in person to participate. Our Information Sheet told participants that the broad purpose of our study was to evaluate the implementation of VR as a tool to measure interpersonal relationships and behavior. Further to discovering an incident of adverse effects in Study 3, we now asked participants in the desktop conditions to report any of the same adverse effects as in the HMD condition. All other aspects of the procedure were unchanged from Studies 2/3. The setup for each modality was in a separate room, so one session of each modality could be run at the same time and conditions allocated as needed.

### Results

A mixed ANOVA with between-subject factor of modality (HMD-based VR vs Desktop) and within-subjects factor of trustworthiness was carried out for each of the dependent variables in both the Door Game and Wayfinding Task. As for previous studies, we corrected for multiple comparisons between ANOVAs through adjusting our α value in the Door Game to .025 and in the Wayfinding Task to .0125. Within each ANOVA here and for the Wayfinding Task, *p* values used for comparisons within families (combinations of modality and dependent variables) described in post-hoc descriptive statistics were adjusted using the Holm-Bonferroni method.

#### Door Game

A mixed ANOVA indicated a significant main effect of trustworthiness on reaction times (F(1,67) = 16.69, *p* < .001, ɳ_p_^2^ = .199; trustworthy M = 1014.42ms, SD = 432.94, untrustworthy M = 1157.77ms, SD = 400.69). However, there was no interaction between trustworthiness and modality (F(1, 67) = 0.98, *p* = .33, ɳ_p_^2^ = .014). There was no main effect of modality, F(1,67) = 0.24, *p* = .623, ɳ_p_^2^ = .004.

A mixed ANOVA also indicated a significant main effect of trustworthiness on following advice (F(1,67) = 93.22, *p* < .001, ɳ_p_^2^ = .582). Additionally, there was a significant interaction between trustworthiness and modality (F(1, 67) = 7.65, *p* = .007, ɳ_p_^2^ = .102). While the frequency of following advice from trustworthy characters in the group who were subsequently to undertake the Wayfinding Task in the Desktop modality (M = 14.84, SD = 3.83) was higher than for untrustworthy characters (M = 10.41, SD = 4.06; *p* < .001, 95% CI = [1.873,7.002]), descriptives indicate that the frequency of following advice from trustworthy characters in the group who were subsequently to undertake the Wayfinding Task in the immersive VR modality was even higher (M = 15.84, SD = 3.01) in comparison to untrustworthy characters (M = 7.84, SD = 3.92; *p* < .001, 95% CI = [5.615,10.385]). When comparing the simple effect of modality at each level of trustworthiness, there was an effect for the untrustworthy characters (*p* = .010, 95% CI [0.170,4.967]), but not for the trustworthy characters, *p* = .269, CI = [-3.392,1.404]. However, the main effect of modality was not significant, F(1,67) = 1.60, *p* = .210, ɳ_p_^2^ = .023.

#### Wayfinding Task

**Fig 7:**
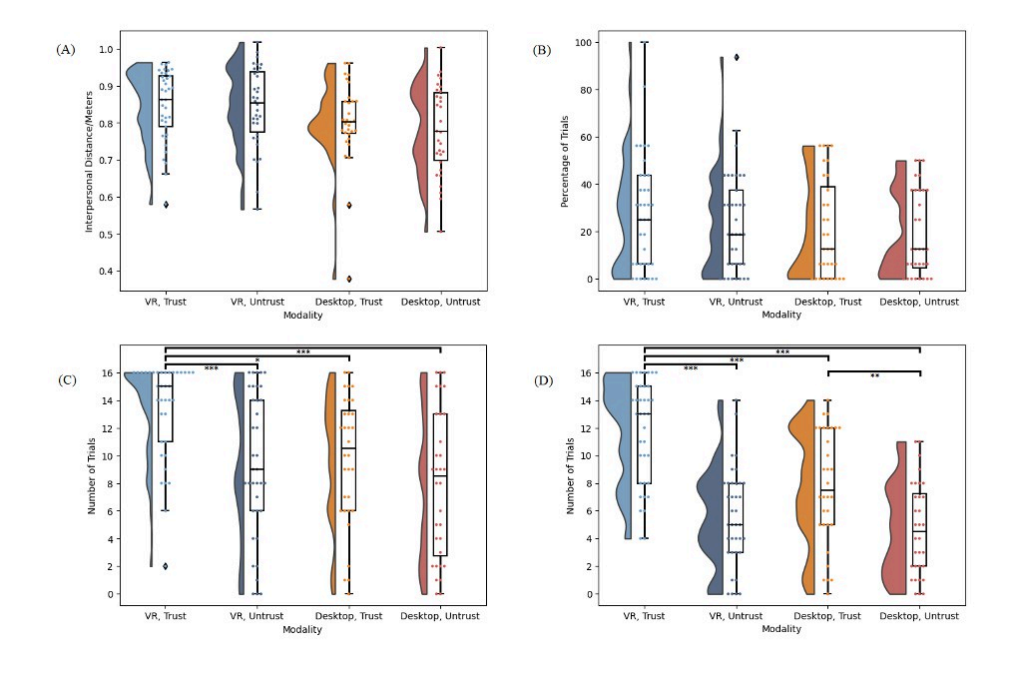
Study 4 data. (A) Distribution of the mean interpersonal distances per participant between the participant and each character on asking for advice, for both the immersive VR and Desktop modalities. (B) Distribution of the percentage of trials per participant on which a given character was asked for advice first. (C) Distribution of the frequency at which each character was asked for advice overall. (D) Distribution of the frequency at which each character’s advice was followed. For all panes, the responses of individual participants are represented by dots. Boxplots show the median and interquartile range for each dataset. * *p* < .0125, ** *p* < .01, *** *p* < .001.

Fig 7 presents the data for all dependent variables in the Wayfinding Task across the desktop and VR modalities in Study 4.

The mixed ANOVA indicated no effects of trustworthiness on interpersonal distance between participants and each character, nor an interaction between trustworthiness and modality (all F < .598, all *p* > .442). However, there was trend towards a main effect of modality on interpersonal distance, F(1,62) = 4.87, *p* = .031, ɳ_p_^2^ = .073. Descriptives (see Table 2) indicated that the interpersonal distance in meters was lower for Desktop (M = 0.79, SD = 0.12) than in immersive VR (M = 0.84, SD = 0.10).

**Table 2:** Study 4 descriptives.

Dependent Variable	VR/Desktop Modality	Trustworthiness	N	Mean	SD
**Interpersonal Distance/meters**	**VR**	**Trustworthy**	34	0.844	0.099
		**Untrustworthy**	34	0.843	0.109
	**Desktop**	**Trustworthy**	30	0.802	0.115
		**Untrustworthy**	30	0.780	0.121
**Trials each character was asked first/percentage**	**VR**	**Trustworthy**	37	27.534	24.450
		**Untrustworthy**	37	23.649	21.607
	**Desktop**	**Trustworthy**	32	20.898	20.857
		**Untrustworthy**	32	18.945	18.064
**Trials each character was asked overall/frequency**	**VR**	**Trustworthy**	37	13.378	3.523
		**Untrustworthy**	37	9.270	5.242
	**Desktop**	**Trustworthy**	32	9.719	4.658
		**Untrustworthy**	32	7.875	5.339
**Trials each character’s advice was followed/frequency**	**VR**	**Trustworthy**	37	11.811	3.770
		**Untrustworthy**	37	5.595	3.492
	**Desktop**	**Trustworthy**	32	7.750	4.143
		**Untrustworthy**	32	4.688	3.355

A mixed ANOVA indicated no effect of trustworthiness nor of modality on which character was asked for advice for advice first, nor an interaction between trustworthiness and modality (All F < .1.73, all *p* > .193).

The ANOVA indicated a significant main effect of trustworthiness on the frequency of approach, out of 16 trials (F(1, 67) = 24.63, *p* < .001, ɳ_p_^2^ = .269), and a trend towards an interaction with modality (F(1,67) = 3.56, *p* = .063, ɳ_p_^2^ = .051). Descriptives indicated that in the immersive VR modality, frequency of approach was higher for the trustworthy (M = 13.38, SD = 3.52) than the untrustworthy character (M = 9.27, SD = 5.24; *p* < .001, 95% C.I. = [1.887, 6.329]), and that the same trend occurred for the Desktop modality (Trustworthy M = 9.72, SD = 4.66; Untrustworthy M = 7.88, SD = 5.34), although this simple effect was not significant (*p* = .237, 95% C.I. = [-0.544, 4.232]). The main effect of modality was also significant, F(1,67) = 6.784, *p* = .011, ɳ_p_^2^ = .092. Descriptives indicated that the frequency of approach was lower for Desktop (M = 8.80, SD = 5.06) than in immersive VR (M = 11.32, SD = 4.89).

Finally, the ANOVA indicated a significant main effect of trustworthiness on following advice (F(1,67) = 59.77, *p* < .001, ɳ_p_^2^ = .471). Additionally, there was an interaction between trustworthiness and modality (F(1, 67) = 6.90, *p* = .011, ɳ_p_^2^ = .093). While the frequency of following advice from trustworthy characters in the Desktop modality (M = 7.75, SD = 4.14) was higher than for untrustworthy characters (M = 4.69, SD = 3.36; *p* .003, 95% CI = [0.673, 5.452]), the frequency of following advice from trustworthy characters in the immersive VR modality was even higher (M = 11.81, SD = 3.77) in comparison to untrustworthy characters (M = 5.60, SD = 3.49; *p* < .001, 95% CI = [3.994, 8.438]). When comparing the simple effect of modality at each level of trustworthiness, there was an effect for the trustworthy characters (*p* < .001, 95% CI = [-6.541, -1.670]), but not for the untrustworthy characters (*p* = .311, 95% CI = [-3.297, 1.483]). The main effect of modality was also significant, F(1,67) = 14.14, *p* < .001, ɳ_p_^2^ = .174. Descriptives indicated that the frequency of following advice was lower for Desktop (M = 6.22, SD = 4.05) than in immersive VR (M = 8.70, SD = 4.78).

#### Implicit Association Test

One participant was excluded as they answered incorrectly on their first attempt on over 40% of trials within a block.

A Wilcoxon signed rank test showed that D-scores from the Desktop modality (M = 0.18, SD = 0.45) were significantly greater than 0, t(30) = 2.24, *p* = .016, d = 0.40. Similarly, a one sample t-test showed that D Scores from the VR modality (M = 0.19, SD = 0.55) were significantly greater than 0, t(36) = 2.16, *p* = .019, d = 0.36. This indicates that participants were faster at the congruent task, suggesting that our trustworthiness manipulation was successful.

An independent samples t-test to compare the Desktop to the VR group showed no difference in D scores, t(66) = -0.097, *p* = .923, d = -0.02.

#### Post-test questionnaire

In terms of adverse effects, of the immersive VR group, six participants reported motion sickness, four reported queasiness, four reported headaches, three reported eye strain and one reported “slight disorientation”. Multiple effects were co-occurring in the same individuals, so these affirmative reports were split across eleven unique participants. This is an adverse effect rate of 29.73%. Of the Desktop group, three reported motion sickness, two reported queasiness, one reported headaches and three reported eye strain, of five unique participants. This is an adverse effect rate of 15.63%. Of the 45 participants who responded with numerical data regarding the size of their personal bubble, the mean estimated size was 91.29cm (SD 71.21). Other responses included ‘a bit’, ‘decent’, or variations thereupon. One participant said it depended on how close they are with the person, and another said it felt like theirs was different in immersive VR compared to in-person interactions. In terms of experience with VR headsets, of 63 respondents, 20 participants in the immersive VR group responded that they had never used a HMD before (14 in the Desktop group), 15 in the VR group had used it 1–2 times (nine in Desktop), one in VR had used it 1–2 times a year (three in Desktop) and only one in VR used it on a monthly basis (zero in Desktop).

### Discussion

Study 4 indicated an effect of trustworthiness on advice following in both the desktop and immersive VR conditions, supported by our Door Game and IAT analysis. This is again in line with what we were expecting, and is similar to the results of Study 2, which first introduced the Door Game to an audience of HMD users. An effect of trustworthiness on frequency of approach was observed, although the simple effect only reached significance in the in the HMD group.

Our Door Game and IAT showed positive results on all measures, in contrast to Study 3. However, it is unclear as to whether the results of Study 4 corroborate our explanation for these differences, as we suggest that the results of Study 3 could be due to self-selection for interest in psychology studies rather than specialist interest in VR, and there are no means to tell what participants’ motivation for joining this study was (which could include interest in VR, or interest in psychology studies).Additionally, the recruiting pool was different, as this was advertised to be an in-person study. However, our ‘experience with headsets’ measure in the post-test questionnaire indicated that the majority of participants likely had little to no experience with VR. In the absence of conclusive information, the results of the IAT in Study 3 merit further investigation. But as discussed in Study 3, we would expect that the Door Game remains the principal confirmatory measure. Overall, this is further evidence for the success of our trust manipulation.

Through our Door Game, we also observe an expected effect of trustworthiness, though with an unexpected interaction for following advice between trustworthiness and testing modality, driven by differences in advice following for the untrustworthy character. As the Door Game took place before participants were aware of their testing modality, it is possible this interaction reflects a false positive, but it is also important to explore other potential causes of this result. Participants were assigned randomly to either modality group, so it is unlikely that this effect resulted from experimenter error as researchers rotated between testing both groups. However, there is always a potential chance of introducing artefacts which may influence participants’ experience through random allocation. Participants were not aware of their group allocation until beginning the Wayfinding Task, although both conditions did occur in separate rooms to maintain the possibility of recruiting in parallel. Thus, this effect is likely the result of random artefacts or differences in the setting, regardless of both being testing labs of roughly the same size though one did visibly contain the headset on arrival; as such perhaps confirming group allocation increased engagement with this early, pre-VR task in the HMD group. Here it is worth emphasizing that in the Door Game, no difference was found between rates of following the trustworthy character across modalities; and ultimately that this difference in rates of following the untrustworthy character’s advice did not carry on to the Wayfinding Task, where the trustworthy character’s advice was followed at a higher rate than untrustworthy across both modalities, and the VR condition’s trustworthy character was followed at a higher rate than Desktop trustworthy also (see Fig 7). In summary, we saw an inverse pattern in comparisons between modalities from the Door Game to the Wayfinding Task, while the effect of trustworthiness on following advice was conserved. This lack of main effect of modality in the Door Game also suggests that any interaction with modality did not affect our results further. It may be worthwhile for future studies to replicate this comparison to explore the potential for confounds.

Our adverse effect rates were highest in the HMD group compared to our previous VR studies. This is to be expected given that this group is the least experienced with VR, as evidenced by our rates of headset usage. None of these effects were severe enough for the participant to warrant ending the experiment early, so all were counted for analysis. However, the large number of effects reported should caution interpretations of the findings. We also formally observe a higher rate of adverse effects in the HMD compared to the desktop group, which is in line with what we expect from the literature [62] and the results of Study 3.

While both desktop and HMD setups continue to demonstrate the suitability of the Wayfinding Task to measure trustworthiness (in line with Study 2 and 3), here we compared two groups from the same recruitment population to observe potential differences in performance. It appears from our analysis that while the effect of trustworthiness on endorsement (advice following) is preserved in both setups, the effect of trustworthiness is stronger in HMD-based immersive VR, with a stronger effect on advice following and a trend towards a stronger effect on approach behavior. As our goal with the Wayfinding Task is to create a socially salient environment for measuring trust, this may mean immersive VR offers distinct advantages in replicating this type of scenario, in line with established theory [40] and suggesting an improvement in terms of effect from Study 3. However, it is also important to consider that we did not formally assess the extent of participants’ experience with HMDs for group allocations within this study; instead assuming that random allocation of participants to groups would suffice to prevent any previous HMD-based VR experience from impacting our results. It may be important for future research to perform such assessment and distribute participants with previous experience across each group accordingly. Nevertheless, when taken together these studies support the use of the Wayfinding Task as a valid tool to measure trust using different testing modalities. However, it may be important to employ and analyze desktop variations with a greater degree of caution than one may otherwise expect from experiments using HMD-based VR.

## General discussion

In this paper we have introduced a variation of Hale et al. [24]’s virtual maze task as a Wayfinding Task and tested its ability to measure trusting behavior in a combination of remote unsupervised, in-person supervised, VR (HMD)-based and Desktop settings. Our data indicate that the new Wayfinding Task is sensitive to manipulations of trustworthiness in all settings. Each study demonstrated that our intended trustworthy character had their advice followed more frequently and also indicated that some form of approach behavior (either who was approached more frequently, or first) was also sensitive to the trust manipulation.

Our design here is based on the ask-endorse paradigm [33, 34], and in particular, Hale and colleagues’ behavioral maze [24]. Hale’s design was particularly attractive in that it introduced a method of measuring trust in adults through a purely behavioral metric, thus addressing many of the issues surrounding explicit declarations of trust which do not reflect ecologically valid scenarios for social interaction. Here, we iterate on this concept in two ways; principally, by developing the design of this paradigm using our Wayfinding Task. This design is similar to Hale in that participants approach a forked path, and are able to consult characters for advice on which path to travel. Instead of having these paths be closed rooms, we designed our Wayfinding Task to resemble an ecologically valid scenario more closely, of navigating an unfamiliar town. This also allowed us to integrate our characters as part of the environment. Secondly, from Study 2 onwards, we provide greater ecological validity through the manipulation of trust using a behavioral paradigm similar to the maze; the Door Game [37]. By introducing this system of manipulating trust through behavior, we aimed to remove the explicit declarations of trust which reduce the ecological validity of manipulating trust through classical tasks such as the investment game. These explicit statements do not gauge predictability [1, 30] and conflate with economic strategy [32], making them less suitable for comparisons to everyday trust interactions. We also hope that, by allowing participants less time to reflect on how they are trusting an individual through declaration of these value judgements and involvement in a cognitively demanding task (the decision making of the Door Game), that they would hence be less susceptible to response biases and that our manipulations would focus more on the relative aspect of trustworthiness across our characters, making our measure (the Wayfinding Task) more purely related to trust.

We also compare recruitment methods and modalities for examining trust in our Wayfinding Task. We examine our results in cohorts of participants obtained via remote recruiting (Studies 1–3) and in-person (Study 4). As the immersion effect of VR can be expected to strengthen ecological validity [39], we also expected a stronger effect in VR compared to a Desktop setup. These effects are observed in our dependent variables. While we frequently observed trends in our ‘asking’ variables (the frequency at which characters were asked for advice, and who was asked for advice first), the strongest effect was consistently seen in ‘endorsement’ (whose advice was followed). These were consistent throughout our studies, and in a direct comparison was stronger in our VR compared to our Desktop modalities. We also only observed an effect on our novel outcome measure, interpersonal distance, in Study 1. However, due to the consistency of our principal measure and its corroboration with data from the Door Game, we argue that these studies show a successful implementation of our Wayfinding Task as a measure of trust. We now go on to discuss these findings in further detail.

In interpreting our results, we may first reflect on our development of characters during Stimulus selection. As our characters were matched on ratings of trust and to appear in the neutral range of our 1–100 scales, and since we observed results with the trustworthy identity being counterbalanced across both characters, we believe this selection criteria sufficient to control for the effects of facial and vocal cues on trustworthiness. However, this does not disregard the possibility of noise being introduced from a variety of factors. In terms of our design, we used only 15 participants in the stimulus selection and did not account for a range of cultural influences that could affect preconceptions of trust. We attempted to account for this by matching the stimuli used in our selection on demographic (female, white, and plain-clothed), which would match any interference effect from participant demographic, like gender or culture [46, 47], across both of our characters. However, perceptions of these categories in our characters may also differ. Further, this may extend to the voices we have used in this study. Both were again matched on demographic (female, Southern English), but this does not exclude the possibility of inferences being made regarding trustworthiness. Additionally, our scenario may introduce other factors than trustworthiness, such as competence, which participants may consider if requesting advice on which direction to follow. This also proposes a methodological challenge to the design of neutral characters, as attempting to control for a wide variety of personality traits through initial percept may result in the removal of more distinguishing features, and hence a lower ecological validity with regards to appearance. As we were successful in obtaining our principal effect (following advice) throughout a counterbalanced design, we argue post-hoc that these selection criteria were sufficient for the current studies, but that future research may wish to develop on this. For example, researchers may wish to employ avatars whose trustworthiness has been manipulated outside of the parameters of an experimental setting, of whom participants may have more stable perceptions of trustworthiness. This could include introducing characters that the participant may already be familiar with, or who they may interact with first in a more ecologically valid trust manipulation.

We did not observe a consistent replication of our findings of interpersonal distance from Study 1. We posit that it may be useful in future to test if this effect is replicable with different methods of manipulation. However, there is also a theoretical basis for the inverse relationship between interpersonal distance and trust which may confound our findings. Rosenberger et al. [36]’s finding that participants stood closer to trusted characters also showed that these distances did not correlate with reports on a trust game. If this is due to the explicit nature of trust reports then we would not expect this relationship in our design, but such postulation is difficult to confirm without the inclusion of explicit reports of trustworthiness, which future studies may wish to investigate. Furthermore, while some studies focused on approaching avatars rate interpersonal distances on average as 38cm [66], results may depend on immersion; if a neurotypical participant experiences fully immersive VR, this can rescale their regulation of interpersonal distance [67]. This is supported by our difference in interpersonal distance across Desktop and HMD conditions in Study 4, and by one participant in Study 4 who answered regarding their bubble that their distance seemed different in HMD VR compared to how it usually does in daily interpersonal interactions. While our design incorporates distances of a similar range to Pochwatko et al. [66], the distance from which participants could interact was capped at slightly over a meter, which may not be enough space for some participants to behave naturally. While this distance was sufficient for the mean estimate of personal bubble across all studies (M = 85.90cm), our mean plus standard deviation is in excess of 1 meter (SD = 59.87cm). We may be able to achieve more representative data if we were to ensure our question resulted in quantitative responses, or if participants were to assess based on visual examples of personal space instead. The latter response may provide data more similar to that of Pochwatko et al.’s study. There are also potential differences in personal space according to culture, which may in principle have varied among participants in our study. Our advertisement offered the incentive of Amazon vouchers in British pounds or US dollars only, so we may tentatively assume that the majority of participants in Studies 1–3 were North American or Western European; in which case the latter group have on average a smaller comfortable interpersonal distance [47]. However, in the absence of conclusive demographic information it must be noted that this is postulation. Additionally, in virtual environments, there is an effect of participant gender on interpersonal space [68], but throughout the present studies we have observed the same null effects in a male-majority (Study 2), gender balanced (Study 3) and female-majority (Study 4) population.

Though following advice indicates whether participants trust each character, the approach frequency has previously been suggested to give insight into the type of trust being expressed. Hale et al. [24] use the term ‘generalized trust’ to refer to an individual’s propensity to trust, whereas ‘specific trust’ refers to how much they trust *a particular individual*. Therefore, we would assume specific trust would differ between our two characters, while generalized trust might differ between participants. Hale et al. [24] postulate that the frequency of approach may be a measure of generalized trust as this would reflect how much participants value others’ advice in general, whereas who was approached first would be a comparative measure between our characters and therefore a measure of specific trust. Despite some gender differences regarding trust and trustworthiness [46], our incidental demographic shifts also did not seem to reflect a stable pattern of demographic effects. One male-majority study (Study 1) showed no effect on asking overall, while another indicated an effect (Study 2). Our more gender-balanced study (Study 3) and one of our female-majority groups (Study 4, HMD group) showed an effect, but another female-majority group did not (Study 4, Desktop group).

However, we are particularly interested in specific trust, as this gives a measure of the different level of trustworthiness between our characters, which we aimed to establish through our trust manipulation. In the first three studies, there was a trend towards an effect on asking for advice first, which indicate the principal directionality that trustworthy characters are consulted for advice more frequently. It may therefore be more useful in evaluating the impact of trust manipulations, which aim to confer trust to one character over the other. However, owing to the null result for this measure in Study 4, one may also consider its face validity in a different scenario; if a person was unsure about the first person’s advice, then they may approach the second to confirm whether the first can be trusted. This approach would mean that the character asked second would reflect the trustworthy character. While we attempted to control for information seeking by having both characters give the same advice in 50% of trials, and while we observed some trend towards an effect for asking first, this is an aspect of individual differences which future studies may wish to account for. Other features of asking for advice may also reflect aspects of trust which we did not initially consider. For example, a high frequency of trials in which both characters were asked for advice (data available on OSF) may reflect an aspect of generalized trust, in that participants value the advice of both characters; or additional decision-making, in that participants infer based on both responses who the trustworthy character is. In all studies, these were significantly below 16 trials (all subjected to Shapiro-Wilk test of normality and test chosen as appropriate. For Wilcoxon signed rank test, Study 1, 2, and Study 4 VR and Desktop groups; all *p* < .001, all r = 1.00; for one sample t-test, Study 3; t(29) = 11.80, *p* < .001, d = 2.16), which would reflect that our studies were not predominantly based on generalized trust or that our Wayfinding Task was not driving decision-making on trust, respectively (full data available on OSF). Future studies should do more to explore and disentangle the relationship between features of asking for advice and experimental design, such as through the use of questionnaires. In comparison to these, the endorsement, operationalized here as which character was followed, showed the highest degree of consistency across studies, being a significant effect of trustworthiness and a comparatively large effect size throughout. Therefore, we may continue to view this as a principal measure of trustworthiness in this type of design going forward (as noted in Study 2).

As participants were instructed to ‘explore’ the city, rather than attempt to travel as far as possible, there may have been potential difficulties in understanding the purpose of the task in both Study 1 and Study 2. However, as there is sufficient data across both studies to confirm a relationship between trust and wayfinding behaviors, we argue there is sufficient evidence to claim internal validity. This may, in part, relate to our demographic; as all of our participants in Studies 1 and 2 owned their own VR headsets, they were likely experienced in games which had objective outcomes, such as travelling as far as they could in a maze. Conversely, only 2 participants (both in the first Study) mentioned in the post-test questionnaire that there was no clear objective. We may postulate this was less of an issue in the second study as the Door Game had an explicit objective (to gain points) and was similar in principle to the maze; which should mean people with less experience with games may also assume an objective for the maze task when presented earlier with the Door Game. In Hale et al. [24] participants were instead instructed to exit the maze in as few rooms as possible, which may create a sense of urgency in participants which would encourage the development of new strategies, or lead to the hope that one character offers successful advice for navigation. The use of ‘explore’ in our instructions means that the advice from characters can be integrated without this external pressure, or assumptions related to outcome. We shall continue to monitor feedback in relation to the maze design when employing new samples.

There are limitations on how we interpret our IAT data based on its positioning in our studies. Our structure throughout followed the same order, where participants completed our Trust Manipulation, Wayfinding Task, then our IAT and Post-test Questionnaire. This positioning is deliberate: although the IAT is an implicit measure, it is quite forthcoming in its mentioning of trust as a concept, and so we wait until the Wayfinding Task is complete to avoid priming our participants directly on this concept before being subject to our main behavioral measures, as an effort to limit demand characteristics. This may have conceptual limitations in our interpretation of the IAT data, for example if any interference were to occur between our manipulation and the IAT (as discussed in Study 3) or simple attenuation of effect, making it unsuitable to interpret the IAT as a direct manipulation check. Indeed, the opposite may also be true; if participants were particularly responsive to the Wayfinding Task, there may be a strengthening of effect in the IAT due to post-hoc rationalizations, even if these were not in truth particularly trust-related. Importantly and in contrast, our Door Game data, when assessed in parallel with our principal data of following advice in the Wayfinding Task, seems to consistently indicate successful manipulation of trust. But in the absence of such data for Study 1, this means that our IAT data should be observed as a purely corroborative measure. Future studies may wish to investigate further its implementation in such designs, or the use of further corroborative measures to test the relation of concepts to pro-social behaviors in such Wayfinding Tasks.

Our results from Studies 3 and 4 seem to indicate that our HMD-based, in-person study produces a stronger relationship in following advice when compared to the desktop, in-person study. Remote studies throughout also had lower dropout rates due to poor data when performed by a population experienced with HMD-based VR performing a VR Study, compared to a population of indeterminate experience with running games or similar programs operating on their own desktop devices. We argue that, taken together, this is suggestive of the suitability of the Wayfinding Task to measure trust across all of the designs presented herein. While our effect was weaker for our desktop study, this implementation has the advantage of a lower frequency of adverse effects [62]. Additionally, the style of unsupervised remote work may offer the benefit of self-paced management of adverse effects; although quantification of these benefits may be hard to achieve. However, there are indications that supervised work may be beneficial to the yield of results within a population. We suggest that future work explores remote, supervised study to see if this is indeed the case, and if supervision can aid in the quality of remote data collection.

There have been recent implementations of a similar maze task outside of Hale et al.’s work which we should also briefly discuss. Work by Lin et al. [69] uses a two-door design similar to Hale et al., 2018, where participants are told their objective is to escape. This is distinct from our work in developing an open-plan, city-like design to offer an interpretation of the paradigm with high ecological validity, and in terms of motivation (where our participants are told to ‘explore’). In terms of ecological validity, their design also offers a few instances of non-diegetic UI which are included for the sake of visual clarity for the participants. These include a visible ‘muted/unmuted’ notification above characters’ heads, and highlighting the outlines of interactable doors when the participant moves to interact with them. Additionally, the trust manipulation in this study was the investment game, and we have discussed our rationale for not including this in the present work. As such, we are comfortable distinguishing the design of the Wayfinding Task used in the present study from implementations of the virtual maze in the work we have discussed. However, it is worth noting that Lin et al. [69] did also find positive effects of trustworthiness on following and asking for advice. Future studies may continue to examine the role of different implementations of the ask-endorse paradigm in conjunction with different trust manipulations.

As we are exploring the design of the ask-endorse paradigm more broadly, we may also investigate its scalability as in Study 4. There are unique concerns with remote and HMD-based studies. In particular, the issue of nausea and general comfort with unsupervised work [64] and relating to recruitment, whether obtaining appropriate sample sizes or issues relating to demographic [49]. We may also comment further on attrition as compared to our remote study. Our HMD studies had completion rates of 53.52% (Study 1) and 45.45% (Study 2), while our remote desktop Study (Study 3) had a return rate of 83.72% and our in-person study, Study 4, had 100%. However, taking into account the data lost due to an incorrect number of trials in the Wayfinding Task, Study 3 had a full completion rate of 42.47%, comparable to our VR studies. It is important to again highlight how attrition was operationalized within this paper. Those who did not complete the study were participants who opened the URL sent to them from the Gorilla page and clicked the ‘Begin’ button (thus generating a participation token) without proceeding through all stages of the study, or who were excluded through means of poor data as described. This may have led to ‘false positives’ for attrition in Studies 1 and 2, where the same participants clicked Begin and then closed the study, by accident or on purpose, to open it later. In Study 3, this may have been due to the lack of supervision as a component of our remote recruitment (see Study 3 Discussion). Horton et al. [70] highlight the disparity in attrition between remote an in-person studies as it is also much easier to withdraw, just by closing the experiment window; and that the time investment to ‘try out’ a particular study is much lower when participating remotely, which presents less of an opportunity cost for withdrawal. The authors also highlight how the best way to remove attrition is through providing incentives to continue only after treatment has occurred; something which we accomplished through providing the link to receive payment only once the data had been collected through the other stages on Gorilla. We also followed the ethical guidelines established in this paper by clearly advertising the expected time for completion and the rates at which incentives were paid. Thus, we believe our data attrition is typical for the type of design employed. We reiterate that while there may be unique challenges to collecting data from studies remotely, our methods used within show efficacious results from implementing the Wayfinding Task.

In the design of our study, a major aim was to increase the ecological validity of an ask-endorse implementation through relating our scenario to a real-world setting, building on the work of Hale and colleagues in measuring trust through behavior. Our trust manipulations, particularly the Door Game, also aimed to develop trust implicitly rather than through explicit declarations and value judgements. By giving less of an opportunity for participants to reflect on their judgements, we also claim that this reduces the chance of conscious influences on decision making and works to prevent the introduction of response biases, such as social desirability bias, which influence economic games [71, 72]. We also worked to minimize external cues (during our Stimulus selection); which is key to avoiding anchoring biases in facial or vocal cues [18–21, 41, 42] and by the development of trust over the course of our manipulation tasks.

This work also provides groundwork for further investigation of trust. Researchers may like to expand on additional qualitative measures to interrogate individual differences, such as personality traits, and their effect on trusting behavior, or limiting the asking portion of ask-endorse by using a forced choice method instead (which may further limit participants trying to test reliability during the measure). Speaking more broadly, it will be important for future research to iterate on the implementation of behavioral measures for trust and see whether the effectiveness maintains in an environment where the trust manipulations and any confirmatory measures are also more ecologically valid, for example by having the manipulation occur in-person or as part of a VR scenario alongside the ask-endorse task.

## Conclusion

In the present paper, we have described a new Wayfinding Task for the measurement of trusting behavior and tested its efficacy with both explicit and implicit trustworthiness manipulations. We observed an effect in both immersive VR and desktop environments on our principal behavioral measures (the frequency of following a trustworthy character’s advice, and approach behaviors). However, there was most frequently a null result for interpersonal distance as a measure for trust. As predicted by Hale and colleagues, there is indeed a stronger effect for HMD-based designs compared to desktop implementations. Finally, remote testing showed higher attrition rates, but similar results on measures of interest, compared with supervised in-person setups. This indicates that paradigms like the Wayfinding Task may be suitable for remote administration.

## Supporting information

S1 File Fact sheet. This is a transcript of the fact sheet administered as our Trust Manipulation in Study 1.
(PDF)

S2 File Door Game instructions. This is a transcript of the instructions used to introduce the Door Game to participants.
(PDF)
