# Supplementary material for: Measuring trust with the Wayfinding Task: Implementing a novel task in immersive virtual reality and desktop setups across remote and in-person test environments
Source: PLoS One. 2023 Nov 28;18(11):e0294420. doi: 10.1371/journal.pone.0294420 (PMC10683989; doi:10.1371/journal.pone.0294420)
Supplement: S1 File — This is a transcript of the fact sheet administered as our Trust Manipulation in Study 1. (PDF) [file pone.0294420.s001.pdf]

Fact sheet

Below the line is a transcript of the items presented for our Fact Sheet. The example below is for Anna as the trustworthy character and Beth as the untrustworthy character. For the opposite condition, the stimuli were identical except for the names being switched.

This is Anna, a character that you will meet in the VR wayfinding task. Click 'Next' to learn more about her.

What did Anna do at University?	When there were class discussions, she always contributed her own ideas and credited others for theirs.
What did Anna do at University?	She graduated with a 2:1 and is still friends with many people from her course.
What does Anna do for Anna living?	She works at a pharmaceutical company that she had an internship at whilst at university.
What does Anna do for Anna living?	She then applied for a graduate role which she was offered.
What does Anna do for Anna living?	As part of her role, she is set a task at the beginning of every month to work on independently.
What does Anna do for Anna living?	She has consistently completed these tasks before the deadline, and to a great standard.
What do Anna's colleagues say about her?	"She had once forgotten her wallet when we were getting lunch at the staff canteen so I brought her food. She paid me back the next day and brought me some chocolates as a thank you!"
What do Anna's colleagues say about her?	"I often confide in her, and she has never discussed my issues with others."
What do Anna's colleagues say about her?	"She has always stepped in when I have needed her. Last year I went on holiday for 3 weeks and entrusted her with my housekeys so that she could water my plants. When I got back, the plants were looking great!"
Employer Reference	"She has been with the company for two years now and is a real asset to the team a great to work with. She is always willing to provide reliable feedback to her colleagues when they need it. When she has an idea that she is excited about, she will stay late to work on it I can rely on her to lock up the lab when she leaves."
What did Anna do last weekend?	She had a long week at work but promised her friends she would go out to dinner, so she kept the promise despite being tired. She had an enjoyable time, ate pizza and laughed with her friends.

This is Beth, a character that you will meet in the VR wayfinding task. Click 'Next' to learn more about her.

What did Beth do at University?	She studied biomedical sciences at Exeter University.
What did Beth do at University?	She took a relaxed approach to her university life and would often skip her morning classes as she preferred to sleep in.
What did Beth do at University?	When it came to group work, she would sometimes forget to complete her allocated tasks, and tended to over-exaggerate her contributions to the project.
What did Beth do at University?	She is still in contact with a few of her friends and graduated with a 2:1.
What does Beth do for a living?	She works as a waitress at her uncle’s restaurant.
What does Beth do for a living?	She likes working for family as she finds it to be a more relaxed environment as she doesn’t get in too much trouble when she is late to her shift.
What does Beth do for a living?	She also receives great tips from customers for her service.
What do Beth’s colleagues say about her?	“She once asked to borrow £50 from me and said she would give it back the next week. It has now been 2 months and she still has not repaid.”
What do Beth’s colleagues say about her?	“I told her that I had a weird rumour being spread about me. The next day I heard her spreading it further and discussing it with the other waiters and waitresses.”
What do Beth’s colleagues say about her?	“We took her out of the Secret Santa group because she didn’t buy a gift when we did it last year, although she had signed up for it.”
Employer Reference	“She has been working at my restaurant for a year now and the customers love her as she always has a nice chat with them about their day. However, her time at my restaurant has not been without issues. Last week she forgot to lock up the restaurant, and I have caught her borrowing money from the tills on more than one occasion.”
What did Beth do last weekend?	She was meant to help her friend move to a new house. The night before, she stayed up late playing video games with some other friends and so missed her alarm in the morning. When she did wake up, she decided that as she was already late, there was no point in going to help with what was left.
