# Supplementary material for: Measuring trust with the Wayfinding Task: Implementing a novel task in immersive virtual reality and desktop setups across remote and in-person test environments
Source: PLoS One. 2023 Nov 28;18(11):e0294420. doi: 10.1371/journal.pone.0294420 (PMC10683989; doi:10.1371/journal.pone.0294420)
Supplement: S2 File — This is a transcript of the instructions used to introduce the Door Game to participants. (PDF) [file pone.0294420.s002.pdf]

Door Game instructions

Below the line is a transcript of the instructions used to introduce the Door Game to participants.

Welcome to the first segment of this study, the 'Door Game'.

In this segment, we will present you with three doors to choose from. When the doors appear, you will have about 5 seconds to pick one door by clicking on it. Picking the 'Correct' door will give you 10 points; picking the 'Incorrect' door will lose you 10 points, and picking the 'Neutral' door will leave your points unchanged.

Your aim is to maximise the number of points you earn, with help from our two characters (pictured below). One of these characters will appear before each set of doors and offer you advice on which to pick. You can choose whether or not to follow this advice. You will automatically move on to the doors after about 5 seconds from being given the advice, or can choose to move by clicking 'Next'.

This segment should take around 5 minutes. When you're ready, click 'Next'.
